# From Cover Crop to Food Crop: Nutritional Composition and Evidence Gaps Limiting Human Consumption of Pearl Millet in Brazil

**DOI:** 10.3390/foods15213782

**Published:** 2026-10-22

**Authors:** Ângela Christina Conte Theodoro, Rita de Cássia Avellaneda Guimarães, João Renato de Jesus Junqueira, Juliana Rodrigues Donadon, Arnildo Pott, Aline Carla Inada, Rosângela dos Santos Ferreira, Karine de Cássia Freitas, Valter Aragão do Nascimento, Priscila Aiko Hiane

**Affiliations:** 1 Graduate Program in Health and Development in the Central-West Region, Medical School, Federal University of Mato Grosso do Sul, Campo Grande 79070-900, Brazil; angela.christina@ufms.br (Â.C.C.T.); inada.aline@gmail.com (A.C.I.); rosangela.ferreira@ufms.br (R.d.S.F.); karine.freitas@ufms.br (K.d.C.F.); valter.aragao@ufms.br (V.A.d.N.); priscila.hiane@ufms.br (P.A.H.); 2 Pharmacy Department, Federal University of Minas Gerais, Belo Horizonte 31270-901, Brazil; jjunqueira@ufmg.br; 3 Pharmaceutical Science, Food and Nutrition Faculty, Federal University of Mato Grosso do Sul, Campo Grande 79070-900, Brazil; juliana.donadon@ufms.br; 4 Graduate Program in Plant Biology, Federal University of Mato Grosso do Sul, Campo Grande 79070-900, Brazil; arnildo.pott@gmail.com

**Keywords:** pearl millet, flour shelf life, functional food, gluten-free, food processing, food security

## Abstract

Pearl millet (*Cenchrus americanus* (L.) Morrone, syn. *Pennisetum glaucum* (L.) R. Br.) is a climate-resilient cereal distinguished from wheat, rice, and oats by a higher lipid content dominated by linoleic acid, essential amino acids, dietary fiber, minerals, and antioxidant compounds. However, its use outside Africa and Asia remains limited; in Brazil it is grown primarily as a cover crop and animal feed, with negligible presence in human diets. This review asks why a nutritious grain produced at this scale fails to enter the human food chain, arguing that its defining nutritional strength is also its main limitation. The lipid fraction triggers rapid hydrolytic and oxidative rancidity post-milling, restricting flour shelf life and impacting downstream processing, formulation, and distribution. We organize the evidence around interacting barriers: sensory acceptance in a population unaccustomed to grain consumption; post-milling lipid instability; nutrient gains and losses across processing, where total and bioaccessible content diverge; the poor dough workability of a gluten-free flour, which leads to blending with other flours and dilutes the attributes that justify its use; and contaminants in pearl millet flour. The challenge is no longer knowing what pearl millet contains, but producing a flour that can withstand distribution, preserve the nutritional properties and gain a place in a food system centered on other crops, a task that requires processing studies, consumer research and evidence generated under Brazilian conditions.

## 1. Introduction

**Figure 1:**
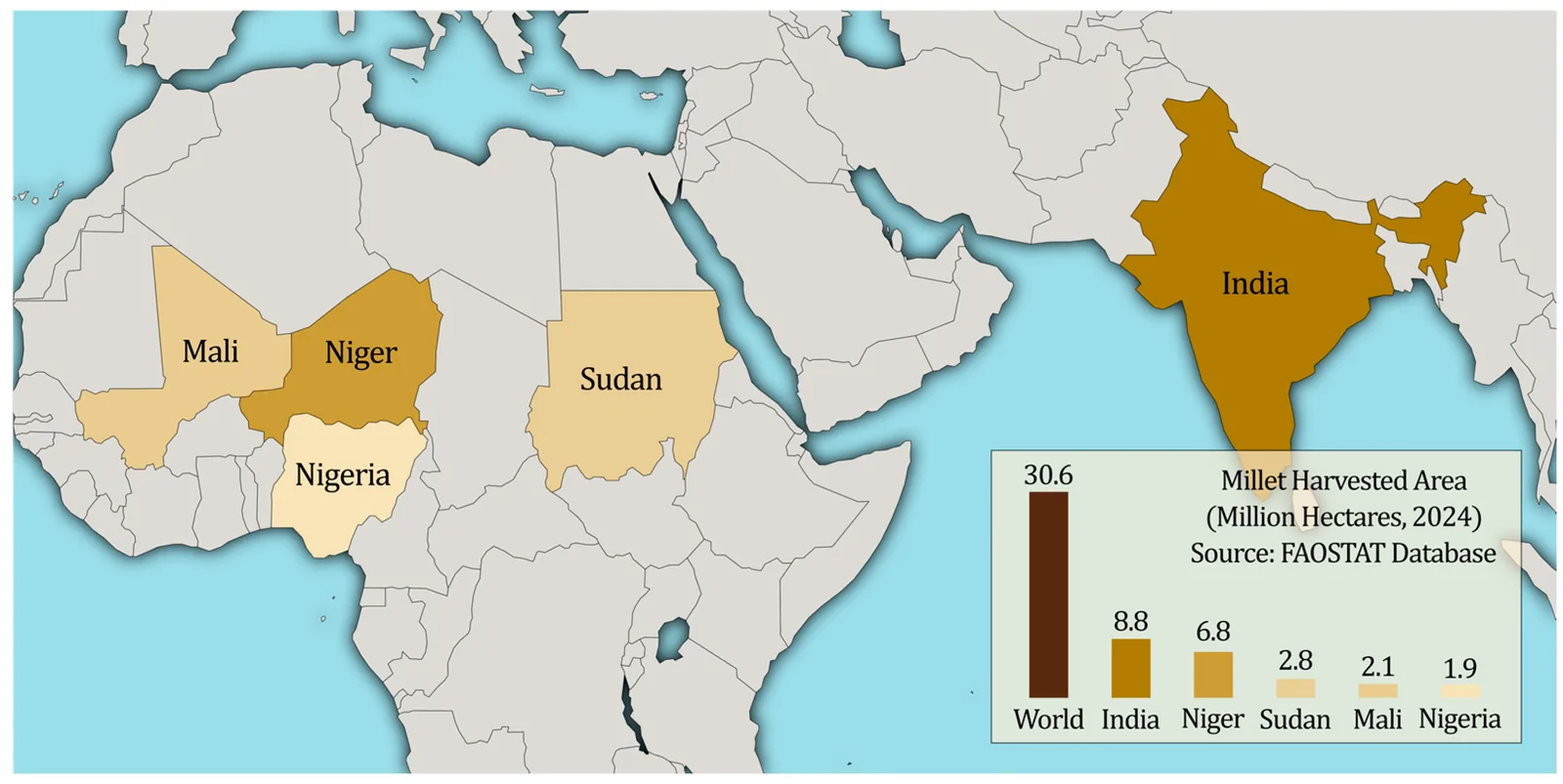
Leading countries in pearl millet harvested area. Source: [4].

Cereals supply a substantial share of the energy and nutrients consumed worldwide, particularly in low- and middle-income countries [1]. Among them, pearl millet (*Cenchrus americanus* (L.) Morrone, also known as *Pennisetum glaucum* (L.) R. Br.), is the sixth most important cereal crop and the most widely grown of the millets [2,3]. In 2024, global production was valued at approximately 9 billion US dollars, with India, Sudan, Niger, Mali, and Nigeria accounting for the largest harvested areas (Figure 1) [4]. As it is a secondary crop in South America, data on harvested areas are decentralized and underestimated. In Brazil, the Brazilian Agricultural Research Corporation (Embrapa) estimates that pearl millet is used as a cover crop on approximately 4 million hectares in the Cerrado region [5]. Its deep root system confers tolerance to water deficit and acidic, low-fertility soils; thus, the crop performs well under adverse conditions and requires comparatively low fertilizer application [6]. These traits have made it a recurring candidate in discussions of climate-resilient food systems.

Pearl millet differs compositionally from wheat (*Triticum* spp.), rice (*Oryza sativa* L.), and oats (*Avena sativa* L.) in its lipid fraction, which is higher than that of major cereals and dominated by linoleic acid [7,8,9,10,11,12]. The grain also provides all essential amino acids, dietary fiber, iron, calcium and zinc, and contains phytosterols, tocopherols, carotenoids and phenolic compounds [8,9,10,13,14,15,16]. It is gluten-free, and short-term studies have reported lower postprandial glycemic responses relative to refined cereals [14,17,18,19,20]. This profile underlies the frequent description of pearl millet as a nutricereal and has motivated a substantial body of reviews on its composition, processing, and functional effects [17,21,22,23].

Although this species has been cultivated in Brazil for at least 50 years and extensively studied [17], its human consumption in the country remains negligible. A grain produced in a region with established milling and food-processing infrastructure has failed to integrate into the human food supply chain; however, the reasons for this have not been systematically analyzed. Previous studies have described the composition and processing techniques of pearl millet, but none have investigated why it is cultivated yet not consumed.

Pearl millet is a cereal with nutritional potential. However, the barriers to its adoption and acceptance in the Brazilian consumer market, including not being included in, and accepted by, the market aimed at Brazilian consumers, have not yet been discussed in terms of criteria in the literature. The first challenge is particularly relevant where pearl millet is not a traditional food. Brazil’s cereal-based food culture is grounded in wheat, and the sensory benchmark for breads, pastas, and baked goods is the wheat matrix. Pearl millet flour differs from this benchmark in two respects. From a sensory perspective, it exhibits a grayish color and notes of bitterness and astringency attributable to phenolic compounds [7,24], characteristics that can intensify as lipid oxidation progresses during storage.

The second challenge in using pearl millet as food is the rapid deterioration and rancidity of the flour after milling. Endogenous lipase hydrolyzes triacylglycerols into free fatty acids (FFAs), and lipoxygenase oxidizes polyunsaturated fatty acids to hydroperoxides, which yield volatile aldehydes and ketones, with peroxidase and polyphenol oxidase contributing secondarily [21,25,26,27,28]. FFAs and peroxide values rise during storage, and the flour typically becomes sensorially unacceptable within two to three weeks [29,30]. Antinutritional compounds impose a third constraint: tannins and phytic acid chelate iron, zinc, and magnesium, thereby reducing their absorption [17,21]. Processing can mitigate both problems, but neither is resolved by the time the flour reaches a domestic or industrial kitchen.

Technologically, the absence of gluten removes the protein network responsible for gas retention and oven rise; consequently, doughs made exclusively from pearl millet flour exhibit reduced volume, inadequate texture, and a shorter shelf life [31]. The practical response, in Brazilian domestic and small-scale production alike, has been to use composite flours, in which pearl millet replaces only part of the wheat or is blended with other flours [32,33,34,35,36]. Blending restores workability and improves acceptance, but it also dilutes the fiber, mineral, and phenolic contributions that motivate the grain’s use in the first place, and it forfeits the gluten-free status that is one of its clearest market advantages. The substitution level at which sensory acceptance and nutritional gain coexist is therefore a central and unresolved question. Finally, flour vulnerability to contamination arises as a critical challenge for the pearl millet value chain, carrying direct implications for global food security, consumer health, and agro-industrial viability [37].

**Figure 2:**
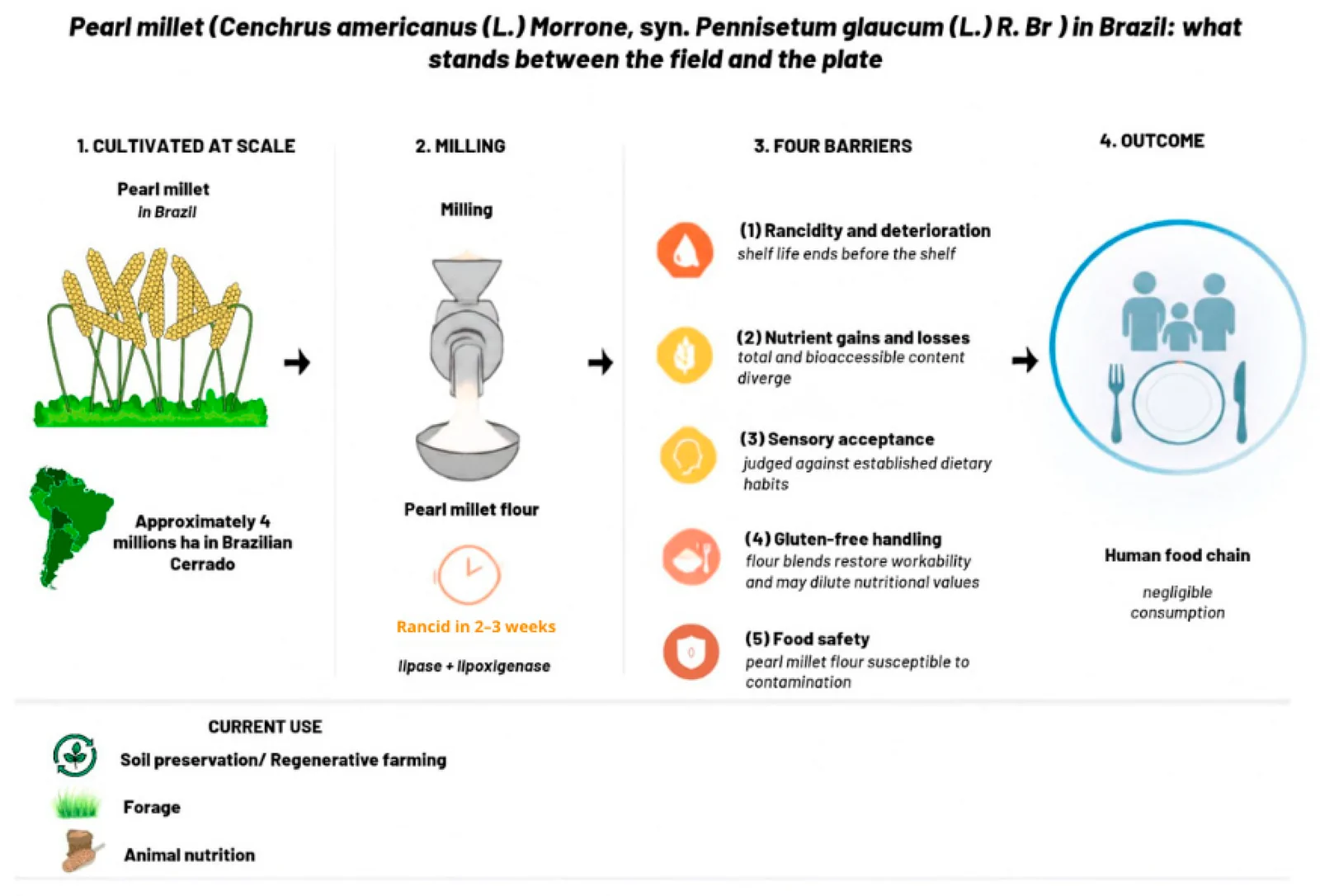
Pathway of pearl millet from cultivation to human consumption in Brazil and the barriers along it.

The FAO designated 2023 the International Year of Millets, renewing global attention to this crop, yet adoption as a human food has not kept pace with production [3]. Previous reviews have covered the composition, processing, health effects, and food applications of pearl millet, including its potential use in Brazil [3,17,21,22,23,26]. This review builds on that literature. It adds an analysis of the path from crop to consumer as a chain of connected steps (field, processing, storage, formulation, and consumer acceptance), in which solving one problem often creates another, examined under Brazilian conditions. The evidence is organized around five interrelated barriers: sensory acceptance in an unaccustomed market; post-milling lipid instability; the difference between total nutrient content and bioaccessibility; technological handling of gluten-free flours, including the dilemma of composite blends; and food security and the implications of flour contaminants. These barriers are then brought together in an integrated analysis from field to consumer (Section 10 and Figure 2), and we conclude by outlining the research and processing priorities needed to move pearl millet from a cover crop to a food crop.

## 2. Materials and Methods

This narrative review does not include quantitative synthesis because the topic spans compositional chemistry, process engineering, sensory science, and nutrition, and the evidence does not fit a single quantitative approach. The search was carried out between January and September of 2026 in Web of Science, MEDLINE (via PubMed), Scopus, SciELO, and Google Scholar. The terms “pearl millet”, “Pennisetum glaucum”, “Cenchrus americanus”, and “Pennisetum americanum” were combined with descriptors related to composition, lipase, lipoxygenase, rancidity, flour shelf life, antinutritional factors, bioaccessibility, composite flour, sensory acceptance, glycemic response, and metabolic outcomes. We also manually checked the reference lists of the retrieved articles and searched Brazilian agricultural and food technology sources. We removed duplicates in Zotero (Version 10.0.6, Digital Scholar, Falls Church, VA, USA) and then checked manually for title variations and abbreviated author names. Figure 1 was created using Inkscape (version 1.4.2, Inkscape Project, www.inkscape.org), and Figure 2 was created using Canva (Canva Pty Ltd., Sydney, NSW, Australia).

Inclusion criteria: We included pearl millet studies that addressed nutritional composition, functional properties, technological processing, or cultural and economic barriers to consumption. We accepted original articles (laboratory composition studies), narrative and systematic reviews on ancient grains, opinion articles, and reports from governments and national or international agencies on the shift in pearl millet from cover crop to food crop. Priority was given to studies with cultivars and edaphoclimatic conditions comparable to those of Brazil, especially those with food technology data. We considered publications in English, Portuguese, and Spanish, preferably from 2014 onwards. We retained older works when they were the primary source of a finding or when no recent data were available. Exclusion criteria: We excluded studies focused only on other millet species or without species identification, studies on animal feed (forage or feed), studies not applicable to the Brazilian agroclimatic context, and publications without full text.

We selected 183 articles. We reported evidence from in vitro assays, animal models, and human studies separately. For composition data, we recorded the sample state (grain, freshly milled flour, or stored flour) whenever available, because pearl millet has high lipid instability.

## 3. Pearl Millet Grains: Nutritional Potential Compared to Other Cereals

The proximate and nutritional compositions of grains and flours presented in Table 1, Table 2, Table 3, Table 4 and Table 5 reflect the natural variability reported in the literature, arising from differences in cultivars, geographical origins, grain fractions (e.g., whole grain vs. refined flour), processing conditions, and analytical methods. Therefore, these tables are intended as a compilation of reference intervals rather than a direct experimental comparison under standardized conditions. The compositions of pearl millet, foxtail millet (*Setaria italica* (L.) P. Beauv.), wheat (*Triticum aestivum* L.), rice (*Oryza sativa* L.) and oats (*Avena* spp.) are shown in Table 1.

Carbohydrates constitute a diverse group of biomolecules, ranging from simple monosaccharides to structurally complex polysaccharides, such as starch and dietary fiber, which are the main source of energy for organisms [42] and are also the predominant component of cereals. Among the cereals described in Table 1, pearl millet and oats contain the lowest quantity of carbohydrates, 68.07 and 70.84 g 100 g^−1^, respectively, while rice presents the highest amount, 79.27 g 100 g^−1^.

Starch is the major component of carbohydrates present in pearl millet (55–75%) [43]. Starch can be classified into three types according to its digestibility by the enzyme glucoamylase in the small intestine: fast-digestion starch, hydrolyzed within 20 min; slow-digestion starch, hydrolyzed between 20 and 120 min; and starch resistant to the digestive enzymes in the small intestine, non-hydrolyzed after 120 min [44,45], being classified as a type of dietary fiber [46].

The term dietary fiber refers to indigestible parts of plants, which the human large intestine ferments all or in part. It is found mainly in cereals, legumes, fruits, and vegetables [47] and is classified as soluble (SF) or insoluble (IF). Microorganisms in the phyla Bacteroidetes and Firmicutes can readily access and metabolize SF in the intestine, producing beneficial functional metabolites such as short-chain fatty acids and phenolic acids [48,49]. Additionally, SF promotes changes in intestinal lumen volume, mass, and viscosity, altering liver cholesterol and lipoprotein metabolism and reducing plasma LDL cholesterol [50].

Insoluble fiber is predominantly present in the structural cell walls and the external tegument of plants. Their hydrophilic properties enable water absorption and volumetric expansion, thereby increasing the volume and viscosity of the food bolus. These effects are associated with greater satiety and faster gastrointestinal transit [51].

Millet grains present a high content of dietary fiber [6,17,18,19,52], on average 12.60 g 100 g^−1^, superior to foxtail millet (7.40 g 100 g^−1^), wheat (2.88 g 100 g^−1^), rice (2.79 g 100 g^−1^) and oats (10.60 g 100 g^−1^) (Table 1), and the highest part of that total is IF. Pushparaj and Urooj [19] evaluated whole-grain flours from two pearl millet varieties, Kalukombu and Maharashtra Rabi Bajra, finding values of 13.3 g 100 g^−1^ and 11.9 g 100 g^−1^ of total dietary fibers, of which 12.6 g 100 g^−1^ and 10.9 g 100 g^−1^ are IF, and 0.70 g 100 g^−1^ and 1.05 g 100 g^−1^ SF, respectively. Ragaee et al. [53] quantified 14.95 g 100 g^−1^ (dry weight) of dietary fibers in whole pearl millet grains, mostly 13.5 g 100 g^−1^ insoluble and only 1.45 g 100 g^−1^ soluble.

Consuming dietary fiber can help control diabetes by adsorbing glucose, modulating the glycemic index and glucose peaks, reducing hyperinsulinemia, improving plasma lipids, and supporting weight control. Water retention, swelling, and viscosity are related to benefits for intestinal health. Fibers also help treat obesity, owing to their capacity for water retention, swelling in aqueous media, oil retention, and cholesterol adsorption, as well as their fermentation, which produces short-chain fatty acids that stimulate enteroendocrine cells to secrete GLP-1 and PYY, thereby promoting diminished hunger and prolonged satiety [54,55,56].

Protein is the second most abundant macronutrient in pearl millet grains, with a mean of 9.33 g 100 g^−1^, higher than in rice (7.49 g 100 g^−1^) (Table 1). Cereals represent the main source of protein derived from plants. High global cereal production has made them a significant source of protein and essential amino acids in the human diet [57].

Proteins are composed of amino acids interlinked by peptide bonds. Amino acids play a fundamental role in maintaining homeostasis and regulating several physiological processes in the human body. These biomolecules act as precursors in protein synthesis and influence essential mechanisms, including gene expression, vasodilation modulation, and the immune response [58]. Some amino acids are essential because the organism cannot synthesize them and must obtain them from the diet [59]. The composition of essential amino acids present in pearl millet and other cereals is presented in Table 2.

**Table 2:** Amino acid composition of pearl millet and other cereals.

Amino Acids (g 100 g^−1^ Protein)	His	Ile	Leu	Lys	Met	Phe	Thr	Trp	Val	Ref.
Pearl millet	2.1–2.8 (2.45)	3.4–4.5 (3.95)	7.5–9.9 (8.75)	2.8–4.0 (3.40)	0.8–1.0 (0.90)	4.1–5.3 (4.70)	2.8–3.9 (3.35)	0.6–1.0 (0.80)	4.5–6.2 (5.35)	[20]
* Foxtail millet	2.1–2.3 (2.20)	4.5–5.1 (4.80)	13.6–16.8 (15.20)	1.5–1.9 (1.74)	3.0–4.0 (3.5)	6.2–6.2 (6.2)	3.6–4.5 (4.05)	ND	5.8–6.3 (6.05)	[60,61]
Wheat	1.9–2.2 (2.05)	3.1–3.3 (3.2)	6.2–6.5 (6.35)	2.0–2.1 (2.05)	1.6	2.4–5.0 (3.70)	2.3–2.5 (2.4)	1.0	4.0–4.2 (4.1)	[18,62]
Rice	2.4–2.5 (2.45)	3.8–4.2 (4.0)	7.7–8.0 (7.85)	3.1–3.7 (3.40)	2.6–2.7 (2.65)	4.9–5.3 (5.10)	3.2–3.3 (3.25)	1.1–1.2 (1.15)	5.3–6.6 (5.95)	[18,63]
Oats	2.50	3.9–4.2 (4.05)	7.6–8.1 (7.85)	4.3–4.4 (4.35)	1.6–1.7 (1.65)	5.4–6.2 (5.80)	2.4–3.5 (2.95)	0.9–1.1 (1.0)	5.4–5.8 (5.6)	[64,65]

* Values are expressed on a dry-weight basis. The ranges (min.–max.) and midpoints reflect the natural compositional variability reported in selected, independent analytical studies. HIS: histidine; ILE: isoleucine; LEU: leucine; LYS: lysine; MET: methionine; PHE: phenylalanine; THR: threonine; TRP: tryptophan; VAL: valine; ND: not determined.

The composition of amino acids in pearl millet can vary according to the cultivar, as demonstrated by Anitha et al. [20]. The grain stands out mainly for its leucine content (8.75 g 100 g^−1^ of protein), which is higher than that of wheat, rice and oats (6.35, 7.85 and 7.85 g 100 g^−1^ of protein, respectively). Nevertheless, foxtail millet presents a higher quantity of leucine (15.20 g 100 g^−1^ of protein) (Table 2). Leucine plays a role in energy homeostasis and lipid metabolism. It stimulates lipolysis and fatty acid oxidation by activating the AMPK/SIRT1/PGC-1α signaling axis. In addition, it modulates the synthesis and/or secretion of adipokines and contributes to the stability of the intestinal microbiota [66].

Regarding the composition of lipids, pearl millet contains 7.04 g 100 g^−1^ on average, superior to that found in other cereals such as foxtail millet (5.18 g 100 g^−1^), wheat (1.23 g 100 g^−1^), rice (0.77 g 100 g^−1^), and oats (4.13 g 100 g^−1^) (Table 1). Lipids provide energy for immediate metabolism and serve as an energy reserve in adipose tissue, which is mobilized during periods of low food intake. Furthermore, they play a fundamental role in transporting and absorbing liposoluble vitamins and provide essential structural and functional functions in the organism [67]. However, their composition matters more than their total quantity, especially the fatty acid profile. The composition of fatty acids present in pearl millet and other cereals is displayed in Table 3.

**Table 3:** Fatty acid composition of pearl millet and other cereals.

Fatty Acids (%)	Pearl Millet	Foxtail Millet	Wheat	Rice	Oats
Linoleic acid (C18:2)	33.05–48.03 (42.49)	65.43–69.53 (67.38)	59.10–66.44 (62.77)	34.72–36.23 (35.47)	38.54–50.69 (44.61)
Oleic acid (C18:1)	26.92–34.93 (30.92)	13.30–13.67 (13.52)	8.45–14.86 (11.65)	39.80–42.23 (41.01)	20.79–40.55 (30.67)
Palmitic acid (C16:0)	17.30–23.05 (19.88)	6.43–8.04 (7.11)	17.51–18.26 (17.88)	16.91–20.64 (18.77)	16.76–20.88 (18.82)
Ref.	[12,13]	[39]	[68,69]	[70]	[68,69]

The ranges (min.–max.) and midpoints reflect the natural compositional variability reported in selected, independent analytical studies.

The composition of fatty acids in pearl millet grains is mainly unsaturated fatty acids, varying between 68% and 77%, depending on the cultivar. These values are composed mainly of linoleic acid (C18:2) [12]. Pearl millet contains, on average, a higher quantity of linoleic acid than rice and is close to the content in oats: 42.49%, 35.47%, and 44.61%, respectively (Table 3). Linoleic acid is generally associated with improvements in cardiovascular health, particularly in lipid profiles, as well as in glycemic control and insulin resistance. A higher ingestion of this fatty acid is linked to a lower risk of cardiac diseases, metabolic syndrome, and type 2 diabetes. However, other metabolic effects remain unclear [71].

Regarding oleic acid (C18:1), pearl millet presents a higher quantity than foxtail millet and wheat, and close to the values described for oats (30.92%, 13.52%, 11.65% and 30.67%, respectively). However, rice presents a higher percentage (41.01%) (Table 3). Oleic acid can benefit mitochondrial and endothelial dysfunction, two mechanisms linked to insulin resistance and type 2 diabetes. Through deacetylation of PGC-1α via activation of the SIRT1–PGC-1α complex dependent on PKA, oleic acid induces the expression of genes that promote fatty acid oxidation. It can also inhibit the expression of E-selectin and sICAM, which are inflammatory mediators [72].

In relation to saturated fatty acids, pearl millet has a mean content of palmitic acid (C16:0) of 19.88%, close to that found in wheat, rice and oats (17.88%, 18.77% and 18.82%, respectively) and higher than in foxtail millet (7.11%) (Table 3). Palmitic acid is a saturated fatty acid that is converted in the cells into phospholipids, diacylglycerol, and ceramides, which trigger the activation of several signaling pathways common to the activation of TLR4 mediated by lipopolysaccharides [73].

Regarding ash content, pearl millet contains, on average, 1.33 g 100 g^−1^, higher than wheat and rice (0.53 g 100 g^−1^ and 0.49 g 100 g^−1^, respectively). Foxtail millet and oats contain higher quantities in comparison with pearl millet, of 1.44 g 100 g^−1^ and 1.77 g 100 g^−1^, respectively (Table 1). Ash refers to the inorganic residue (mineral) remaining after incineration of the organic matter [74]. The literature on the minerals of pearl millet and other cereals presents considerable variation, and, in general, the minerals described are iron, calcium, and zinc. The content of iron, calcium, and zinc present in pearl millet and other cereals is shown in Table 4.

**Table 4:** Mineral composition of pearl millet and other cereals.

Minerals (mg 100 g^−1^)	Iron	Calcium	Zinc	Ref.
Pearl millet	5.03–30.28 (13.91)	22.59–42.75 (30.89)	2.38–4.06 (3.06)	[9,10,18]
* Foxtail millet	3.73–17.74 (10.47)	9.27–30.10 (19.68)	7.37–11.31 (9.34)	[9,75,76]
Wheat	1.18–1.77 (1.47)	19.38–22.00 (20.59)	0.88–3.89 (1.97)	[9,11,18]
Rice	0.14–1.02 (0.58)	4.00–10.93 (7.46)	1.35–1.68 (1.51)	[11,18]
* Oats	2.50–3.00 (2.80)	44.00–102.70 (64.50)	1.60–2.00 (1.88)	[77]

* Values are expressed on a dry-weight basis. The ranges (min.–max.) and midpoints reflect the natural compositional variability reported in selected, independent analytical studies. The ranges (min.–max.) and midpoints reflect the natural compositional variability reported in selected, independent analytical studies.

Pearl millet contains a higher mean quantity of iron than foxtail millet, wheat, rice and oats, 13.91, 10.47, 1.47, 0.58 and 2.80 mg 100 g^−1^, respectively. These values refer to total content; the fraction available for absorption is discussed in Section 9.2. Concerning calcium, only oats have a higher quantity than pearl millet, at 64.50 and 30.89 mg 100 g^−1^, respectively. In addition, the grain contains more zinc than wheat, rice, and oats, at 3.06, 1.97, 1.51, and 1.88 mg 100 g^−1^, respectively, while foxtail millet presents a higher mean content, 9.34 mg 100 g^−1^ (Table 4). These variations may depend on crop location, soil, genotype, and climatic conditions.

Minerals are essential nutrients that support human health by regulating several physiological processes. Their main functions include developing and maintaining bone structure, serving as cofactors in enzymatic reactions, supporting nervous signaling, and modulating the immune response. Maintaining adequate mineral levels through a balanced diet and monitoring vulnerable populations is fundamental to promoting health and preventing disorders associated with mineral deficiencies [78].

## 4. Pearl Millet Grains: The Presence of Bioactive Substances

Bioactive compounds, including vitamins and phytochemicals such as carotenoids, tannins, and phenolic compounds, may promote immunological homeostasis by enhancing anti-inflammatory and immunomodulatory mechanisms and reducing oxidative stress by binding free radicals and neutralizing reactive oxygen species (ROS) [79,80].

Oxidative stress is the leading cause of the onset and progression of major human health disorders, and high concentrations of ROS and reactive nitrogen species damage proteins, lipids, and DNA, thereby increasing susceptibility to chronic degenerative diseases [81].

Pearl millet contains a wide range of bioactive substances, including antioxidants. Among the main compounds present in the grain are phytosterols, tocopherols, carotenoids, and phenolic compounds [8,13,18,27,82]. These compounds can combat, prevent, or control cardiovascular diseases, cancer, type 2 diabetes, and arterial systemic hypertension and improve gastrointestinal health [83]. Table 5 presents the main bioactive substances present in pearl millet and their properties.

**Table 5:** Bioactive substances in pearl millet.

Bioactive Substances			** Potential Biological Effects	Ref.
Phytosterols (%)	Campesterol	20.28–19.40	Antioxidant with potential help in reduction of plasma cholesterol, modulation of inflammatory pathways and reinforcement of the immune system	[13,84]
	β-sitosterol	51.58–53.30		
	Stigmasterol	6.19–6.42		
Tocopherols (%)	α-tocopherol	2.86–3.81	Antioxidant with potential protective effect against cell damage causative of non-transmissible chronic diseases.	[13,85]
	γ-tocopherol	87.32–88.51		
	Δ-tocopherol	0.43–2.16		
Carotenoids (µg 100 g^−1^)		293	Antioxidant, ocular and cardiovascular health, and cognitive function.	[18,86]
* Total phenolics (mg GAE g^−1^)		72.08–136.25	Antioxidant, anti-inflammatory, antiproliferative, antidiabetic and antiaging.	[87,88]
Antioxidant activity (% DPPH)		66.90–84.78	Acts against oxidative stress-mediated pathological processes, neutralizing free radicals.	[88,89]

* Values are expressed on a dry-weight basis. ** The potential biological effects described above are dependent on the dose–response relation, bioavailability, and interaction of compounds in the food matrix. The literature data, in general, are based on in vitro and/or in vivo experimental models using isolated compounds or concentrated extracts; therefore, consumption of whole grains should not be interpreted as direct or absolute therapeutic action.

El Kourchi et al. [13] evaluated the content of phytosterols in pearl millet grains cultivated in two distinct locations. The grains cultivated in Morocco had values of 20.28%, 53.30%, and 6.19% for campesterol, β-sitosterol, and stigmasterol, respectively, while grains cultivated in India had values of 19.40%, 51.58%, and 6.42%, respectively (Table 5). Phytosterols are recognized for their ability to reduce cholesterol, their anti-inflammatory and antioxidant properties, and their benefits in strengthening the immune system. These benefits are attributed to phytosterols’ ability to modulate the immune response, oxidative stress, adipose tissue metabolism, hypercholesterolemia, and intestinal dysbiosis [84].

Regarding tocopherols (Table 5), the grains cultivated in India had higher percentages of α-tocopherol and γ-tocopherol than the Moroccan grains, with values of 3.81% and 88.51%, respectively, versus 2.86% and 87.32%. Nevertheless, the content of Δ-tocopherol was higher in grains cultivated in Morocco (2.16%) than in grains from India (0.43%) [13].

Tocopherols are among the most important liposoluble antioxidants present in foods. Their health benefits are linked to gene expression regulation and antioxidant activity, as they act as hydrogen donors. Thus, they influence metabolism and membrane phospholipid integrity, affecting enzymatic and spontaneous free-radical oxidations and the oxidation of polyunsaturated lipids to form eicosanoids and lipid byproducts, many of which mediate immune and vascular processes and/or act as toxic molecules. They can contribute to the prevention of certain types of cancer, such as breast, gastric, and lung, as well as cardiac diseases, diabetes, and obesity [85,90].

Carotenoids are natural pigments found in most fruits and vegetables and are not synthesized by humans; their ingestion or supplementation is necessary to supply the body. Overall, evidence suggests that carotenoids exert antioxidant effects, support ocular health, and improve cognitive and cardiovascular health. Pearl millet grains contain carotenoids, notably lutein, zeaxanthin, and β-carotene. Lutein and zeaxanthin act to repair photoinduced oxidative damage, while β-carotene has provitamin A function [18,86,91].

The total content of carotenoids and other bioactive compounds in millets varies. Longvah et al. [18] reported 293 µg 100 g^−1^ of carotenoids in the grains, and Mawouma et al. [82] described higher values in flour of two different varieties, 0.52 mg 100 g^−1^ (520 µg 100 g^−1^) for Gawane, and 0.53 mg 100 g^−1^ (530 µg 100 g^−1^) for Mouri (dry extracts). For lutein, zeaxanthin, and β-carotene, the reported values in pearl millet grains are 29.69 µg 100 g^−1^, 9.30 µg 100 g^−1^, and 28.23 µg 100 g^−1^, respectively [18].

The scientific literature reports varied quantities of phenolic compounds for *P. glaucum*. Bouajila et al. [88] evaluated total phenolic compounds in 75 different pearl millet varieties from various geographic regions and grown under diverse agroclimatic conditions and observed a range of 72.08 to 136.25 mg GAE/g (dry weight). Phenolic compounds have the capacity to act against oxidative stress, associated mainly with the activity of eliminating free radicals, which neutralizes reactive oxygen and nitrogen species, contributing to their antioxidant, anti-inflammatory, antidiabetic, antiproliferative, and anti-aging activities [87].

Regarding antioxidant capacity, Bouajila et al. [88] reported inhibition percentages, measured using the DPPH method, ranging from 66.90% to 84.78% across 75 different cultivars. This is because the pearl millet grain contains a wide variety of bioactive substances with antioxidant activity, as previously described.

Despite its varied bioactive composition, pearl millet also contains antinutrients, or antinutritional factors, that can influence its biological activity, bioaccessibility, and bioavailability through interactions with other components of the food matrix, including various bioactive constituents [92]. Antinutrients, or antinutritional factors, are secondary metabolites and other highly bioactive compounds with structure-dependent biological effects that can reduce nutrient bioavailability and affect growth, reproduction, and human health [93]. The main antinutrients in pearl millet grains are phytic acid and tannins [24,94], which are known to interfere with nutrient and micronutrient absorption and affect health [95,96,97]. However, various methods can reduce or inhibit antinutrients in grains.

## 5. Metabolic and Functional Effects

The varied composition of pearl millet’s nutritional profile may confer health benefits in humans. Research in in vivo and in vitro models assessed the impact of the cereal components on anti-obesity, hypolipidemic, hypoglycemic, anti-inflammatory, antisteatotic, and anticancer effects [98,99,100,101,102,103,104]. Table 6 presents the metabolic and functional effects of pearl millet described in the literature.

In a study conducted by Alzahrani et al. [98], obese Wistar rats (*Rattus norvegicus*) were fed a hyperlipidic diet with added powder pearl millet (10, 20 and 30%) and pearl millet ethanolic extract (25, 50 and 100 mg kg^−1^) for 8 weeks. Administering pearl millet powder and ethanolic extract resulted in significant metabolic improvements. They observed a progressive, dose-dependent reduction in final body weight, liver weight, and visceral and epididymal fat deposits, followed by reductions in triglyceride, cholesterol, fasting glucose, and inflammatory markers (C-reactive protein and tumor necrosis factor alpha) levels. Those authors concluded that the grain, either in powder or extract form, exerts anti-obesity, hypoglycemic, hypolipidemic, anti-inflammatory, and antisteatotic activities in a dose-dependent manner.

Theodoro et al. [99] investigated the effects of germinated pearl millet flour (286.3 g of flour kg^−1^ of diet) on inflammation, oxidative stress, adiposity, and hepatic steatosis in Wistar rats submitted to a diet rich in saturated fat and fructose (HFHF) for 10 weeks, after induction of metabolic alterations for 8 weeks. Compared with the HFHF control group, flour supplementation reduced adiposity (32.89 g vs. 24.18 g) and tumor necrosis factor-alpha (TNF-α) concentrations (14.4 pg mL^−1^ vs. 98.80 pg mL^−1^). Also, they observed increased expression of the protein PPARα and total hepatic antioxidant capacity, indicating that germinated pearl millet exerts anti-inflammatory, antioxidant, and anti-steatosis effects, thereby mitigating adiposity and hepatic steatosis.

The potential of pearl millet was investigated in the context of oncological pathologies by Oliveira et al. [100], who assessed the effects of oral supplementation with milled grain added to the diet (concentrations of 29% and 36%) of male *Mus musculus* mice of the lineage FVB/n (10 HPV−, Wild Type and 15 HPV+) for four weeks. In HPV+ animals, the intervention reduced glycemia and optimized antioxidant responses, while attenuating the severity of cutaneous and organ lesions induced by the virus. The results suggest that pearl millet exerts hypoglycemic, antioxidant, and anti-inflammatory effects, positioning it as a food that may be chemopreventive against human papillomavirus.

In a study on the bioavailability of iron, Theodoro et al. [101] evaluated the effect of added pre-cooked flours of whole conventional pearl millet, germinated, and extruded in the diet of Wistar rats by the hemoglobin depletion–repletion method. The concentrations varied by flour type (conventional 267.8 g kg^−1^; germinated 287.8 g kg^−1^; and extruded 212.8 g kg^−1^), with a total of 13.29 ppm iron across treatments. All evaluated groups showed increased DMT1 and hephaestin gene expression, increased serum iron, and reduced transferrin, Nrf2, and TNF gene expression, indicating promising mineral bioavailability.

Vidhyalakshmi et al. [102] measured blood glucose levels and the glycemic index in vivo in healthy and prediabetic participants after ingestion of breads prepared with the starch–germ complex and germ by dry heat treatment (PMSGH) at proportions of 20%, 25%, and 30%. Incorporating 30% PMSGH into the bread reduced the glycemic index from approximately 100 to 52.31 in pre-diabetic participants, a value close to that in healthy participants. Additionally, the complex reduced blood glucose levels in both healthy and pre-diabetic patients compared with white bread (control).

In another study by Nani et al. [103], the phenolic extract of pearl millet was evaluated for its pro-apoptotic effects on osteosarcoma U-2OS cells at concentrations of 20 to 100 µg mL^−1^. The results indicate that these compounds induced dose-dependent cell death via caspase-dependent apoptosis. Additionally, the data showed that phenolic compounds activated the p38 MAPK and SAPK/JNK signaling pathways while attenuating AKT activation and increasing intracellular calcium, resulting in cell cycle withdrawal and apoptosis.

Kaila and Muthukumar [104] assessed the activity of enzymatic inhibition of lipase, α-amylase and α-glucosidase in ethanolic extracts from three varieties of pearl millet at the concentrations of 50, 100, 200, 400, 600, 800 and 1000 µg mL^−1^. The mean lipase inhibition was 76.02%, suggesting probable potential control of obesity. Inhibition of α-amylase and α-glucosidase reached 82.88% and 82.83%, respectively, with possible benefits for diabetic individuals.

The evidence presented demonstrates pearl millet’s potential as a functional food. The studies indicate that either the whole grain or its extracts act in a dose-dependent manner to regulate metabolic disorders, control systemic inflammation, and modulate critical cell-survival pathways. It also showed no toxicity to normal cells. However, although results in vitro and in animal models are promising, the transition to clinical protocols and the establishment of safe doses for humans still require further study.

## 6. Principal Pearl Millet Processing Techniques

Pearl millet processing is usually presented as a set of techniques that improve digestibility, reduce antinutrients, and extend shelf life, and the available repertoire is broad: decorticating, milling, soaking, acid treatment, roasting, parboiling, cooking, extrusion, germination, and fermentation [8,22,105,106,107,108,109,110,111,112,113,114,115,116,117,118,119]. That framing is nonetheless incomplete. Almost every technique reported for this grain removes something of nutritional value while removing something undesirable, and the balance between the two is rarely quantified. Table 7 summarizes the principal methods described in the literature and, for each, the compositional cost that accompanies the reported benefit.

Physical operations primarily remove anatomical fractions that concentrate both antinutrients and micronutrients. Decorticating removes 12–30% of the grain, lowering phytic acid, tannins, and enzyme inhibitors, while simultaneously removing ash, lipids, and fiber [105,126,127]. The effect is dose-dependent: only the mildest decortication extended flour shelf life by reducing lipoxygenase activity and lipid oxidation, whereas more intense treatments increased non-enzymatic lipid oxidation and depleted bioactive compounds [106]. It also alters proximate composition, particularly mineral content, indicating that these components are concentrated in the bran fractions removed [26]. Milling, in turn, redistributes rather than eliminates antinutrients: particle size reduction determines the flour’s physicochemical behavior and the characteristics of the resulting products, and varies with the equipment used [128,129]. Oxalates and phytates concentrate in the bran fraction, so semirefined flour is nutritionally superior in terms of mineral bioaccessibility yet contains less mineral in absolute terms than the whole meal flour it replaces [107].

Physicochemical treatments operate through leaching and therefore carry the same ambiguity. Soaking reduces phytic acid and tannins and increases mineral bioavailability, but it leaches phosphorus, zinc, iron, and manganese; 12 h has been proposed as the optimal compromise [108]. High-pressure soaking reduced phytate by up to 80.6% while increasing free iron severalfold and is among the few treatments reported to improve one side of the balance without a proportional loss on the other [109]. Dilute acid treatment inactivates lipases and reduces antinutrients, but also depletes phosphorus, calcium, and iron, with losses accentuated at 24 h [17,110,111].

Thermal and thermomechanical treatments show the widest spread of outcomes. Roasting improves palatability and digestibility through dry heat at controlled temperature and time [130], reducing tannins and phytic acid under mild conditions; however, higher temperatures degrade phenolic compounds and fiber and raise concerns about acrylamide formation, a heat-induced contaminant well documented in cereal-based foods [19,112,131]. Cooking by immersion, steam, or pressure likewise lowers phytic acid at the expense of total polyphenols, β-carotene, and antioxidant activity [114,115,116]. Parboiling and extrusion address grain stability and product functionality simultaneously and are therefore most relevant to the barriers discussed in this review. Parboiling combines conditioning, cooking, and drying; induces starch gelatinization and retrogradation [113]; and has been reported to extend flour shelf life, improve lipid stability, and enhance milling yield and functional properties [120]. It increased free and bound phenolics in pearl millet porridge and couscous and lowered the expected glycemic index, at the cost of reduced protein digestibility [113].

Extrusion, whose outcome depends on moisture, temperature, screw speed, and pressure [132,133], inhibited lipase and lipoxygenase by roughly 78–79% and reduced phytic acid and condensed tannins by up to 89% and 85%, respectively [119] and promoted amylose–lipid complexes that slow in vitro starch digestion [118]. Notably, extrusion is also the treatment most compatible with industrial scale and with the distribution constraints discussed in this Section.

Biochemical treatments deliver the broadest nutritional gains and the least industrial readiness. Germination breaks seed dormancy and activates amylolytic and proteolytic enzymes over days [134], reducing phytates, releasing bound phenolics, and raising antioxidant activity, while lowering lipid content and altering the mineral profile in both directions [121,122,123]. Fermentation, whether submerged or solid-state [135], increases bioactive compounds and antioxidant capacity and generates hydrolytic enzyme activity, and additionally yields non-digestible oligosaccharides with prebiotic potential while modifying the accessibility of the fiber complex to the intestinal microbiota [136]; reported losses include ash and fiber [124,125]. Both processes require days rather than minutes, and neither has been evaluated at a scale relevant to a commercial flour supply chain.

Two observations follow from this body of work and are central to this review’s argument. First, effects are almost always reported as changes in total content, and almost never as changes in bioaccessible content, so the nutritional consequence of a given treatment is frequently unknown. Second, and more consequentially, the analytical point is rarely stated: most compositional data refer to grain or freshly processed flour, not to flour after a defined storage period. For a matrix whose lipid fraction degrades within weeks, this omission means that the reported nutritional value of a processing method and the value delivered to a consumer are not necessarily the same quantity.

Pearl millet processing involves a trade-off between technological stability and nutritional preservation. Higher processing intensity can promote enzymatic inactivation, reduction in antinutritional factors, and the extension of shelf life, but it can also increase energy consumption and costs, as well as cause losses of nutrients, bioactive compounds, and sensory quality. Therefore, technology selection must simultaneously consider nutrient retention, bioaccessibility, functionality, sensory quality, energy efficiency, cost, and industrial scalability.

Among the alternatives, parboiling and extrusion present potential for industrial applications due to their ability to combine stabilization, reduction in enzymatic activity, and improvement of functional properties. Germination, malting, and fermentation favor the reduction in antinutritional factors and the increase in bioaccessibility and bioactive compounds, although they require longer processing times and offer lower throughput. Non-thermal technologies, such as ultrasound and cold plasma, as well as thermal or combined treatments, including microwave, blanching, hydrothermal treatment, antioxidants, and near-infrared, also show potential to reduce rancidity and extend shelf life, especially when applied in combination [120,137].

However, there is no universally superior technology. The choice must be guided by the product and by the balance between technological performance and nutritional, energetic, and economic impacts. For large-scale flour production, fast, continuous, and easily controllable processes, such as extrusion and parboiling, show greater potential for scalability, whereas germination and fermentation may be more suitable for value-added functional products. The main challenge lies in determining the optimal balance between the minimum processing intensity required to ensure stability and shelf life, while simultaneously maximizing nutrient retention and bioaccessibility, sensory and functional quality, and economic and industrial viability.

## 7. Pearl Millet Food Products

When processed, millet grains yield various products with functional potential; however, the cereal is not yet used on a large scale as a food in Brazil, highlighting the need to study its consumption across different processing methods. Processed pearl millet has been used in breads, biscuits, cakes, fermented beverages, dairy formulations and meat preparations, with generally favorable sensory scores (Table 8). Its water retention and textural properties suit bakery and fortification applications, and the absence of gluten positions it for the coeliac and gluten-free market, whose demand reflects both rising diagnosis and greater consumer awareness [3,13]. Yet the grain remains absent from the Brazilian food supply, and the product literature helps explain why.

As shown in Table 8, products made with pearl millet, whether gluten-free or not, tend to contain bioactive substances such as phenolic compounds, flavonoids, and vitamins, which enhance antioxidant capacity, and higher levels of minerals and dietary fiber, while presenting a lower glycemic index. However, processing type, combined with the specific raw materials used, can influence this composition.

Pessanha et al. [138] developed gluten-free whole-grain breads using raw and extruded pearl millet flours, both individually and in blends, and the results indicated that the bread made with extruded flour showed increased antioxidant capacity (from 69.1% in the bread made with raw flour to 80.3% in the bread made with extruded flour) and phenolic compound content (122.4–131.9 μmol GAE g^−1^), as well as improved anti-hyperglycemic activity (90.3–99.2%), compared to the bread made with raw flour. Marinho et al. [139] reported similar results for breads made with raw, extruded, and germinated pearl millet flours using the straight-dough and sponge-dough methods, compared with commercial products and those made with refined wheat flour.

Among bakery products, biscuits and cookies are popular and affordable items. Ramashia et al. [34] evaluated the composition of biscuits made with varying levels of wheat flour substitution using malted pearl millet flour and orange peel flour and reported a proportional increase in ash and fiber content (4.50–5.54% and 11.44–16.24%, respectively) corresponding to the increase in substitution (60% wheat flour, 32% malted pearl millet flour and 8% orange peel flour). Furthermore, the authors reported significant increases in total phenolic compounds (1.40–3.56 mg GAE 100 g^−1^), total flavonoids (2.91–6.79 mg QUE 100 g^−1^), and antioxidant capacity (1.78–8.64 mg GAE g^−1^). Kulkarni, Sakhale, and Chavan [35] reported similar results for cookies made with varying levels of pearl millet flour as a substitute for wheat flour. Ash content increased from 0.9 to 1.7 g 100 g^−1^, and fiber content rose from 0.15 to 1.14 g 100 g^−1^ in cookies made with 100% pearl millet flour compared to the control (100% wheat). In biscuits prepared using pearl millet flour washed in distilled water or an alkaline solution (0.05 M NaOH), increases were reported in total phenolics (from 1.90 to 4.72 and 4.33 mg gallic acid g^−1^ sample, respectively) and antioxidant capacity (from 10.39 to 15.85 and 14.52%, respectively) [36]. Other studies also cite the distinct composition of biscuits and cookies when pearl millet is added [140,141].

Pearl millet is also used in beverages. Kunu is a beverage produced from pearl millet grains that are hydrated, germinated, ground, and fermented, with spices added. The beverage contains 9.9% protein, 1.77% ash, and 3.70% fiber. Its mineral content includes calcium (182.37 mg 100 g^−1^), magnesium (145.90 mg 100 g^−1^), potassium (12 mg 100 g^−1^), phosphorus (10.50 mg 100 g^−1^), and trace amounts of iron and zinc (0.30 and 0.50 mg 100 g^−1^, respectively) [142]. In another study by Atosona et al. [143], an instant pearl millet and baobab (*Adansonia* spp.) beverage was found to contain minerals such as iron (18.91 mg 100 g^−1^), magnesium (188.65 mg 100 g^−1^), calcium (512.90 mg 100 g^−1^), potassium (1033.20 mg 100 g^−1^), and phosphorus (339 mg 100 g^−1^), as well as citric acid (320.30 mg 100 g^−1^) and vitamin C (27.70 mg 100 g^−1^).

Pearl millet grains have also been tested in dairy and meat products. Samtiya et al. [144] evaluated a functional fermented beverage based on milk and pearl millet that contained 3.65% protein and 1.39% ash, notably featuring iron (7 mg L^−1^), zinc (6.44 mg L^−1^), calcium (1023 mg L^−1^), and phosphorus (243.60 mg L^−1^) contents, as well as stability for one week under refrigeration (4 °C). Roasted pearl millet flour was utilized in a probiotic strained yogurt-like dessert at concentrations ranging from 2.5% to 8%, along with the addition of powdered sugar (25–45%) and hibiscus extract (2.5–8%). The formulation optimized via Design-Expert software, comprising 4.86% pearl millet, 4.4% hibiscus extract, and 29.47% powdered sugar, achieved a probiotic viability of 7.53 log10 CFU g^−1^, exceeding the recommended threshold, alongside an increase of 26.94% in protein content and 11.39% in mineral content, in addition to a shelf life of 28 days at 4 °C [145]. An increase in protein and mineral content was also reported by Patel et al. [146] in a functional goat-milk-based nutritional supplement enriched with fermented pearl millet and finger millet flours. However, the authors noted a decrease in vitamins A, D, E, K, and B-complex levels compared to the commercial reference product (malt-based nutritional beverage powder). In yogurts enriched with malted pearl millet flour (70:30), the authors reported increased levels of manganese (0.35–0.77 ppm) and iron (1.5–6.09 ppm) compared with the control (plain yogurt) [147]. In a meat-based study, Brasil et al. [148] prepared kibbeh using hydrated or roasted pearl millet flour; the resulting products exhibited good oxidative stability even after 90 days of frozen storage.

Other products, such as cakes, waffles, and pasta, were developed and evaluated. Costa et al. [33] evaluated the quality of different cake formulations made with extruded pearl millet flour supplemented with various fiber sources (wheat bran, oat bran, rice bran, or psyllium). Fiber content ranged from 4.65% to 6.10%, and all formulations were considered fiber sources. In waffles, a blend of wheat, finger millet, and pearl millet flours (40:40:20) increased the content of total phenolic compounds (7.53–39.79 mg GAE 100 g^−1^ dry basis) and total flavonoids (5.46–11.44 µg QE g^−1^ dry basis) compared to the control made exclusively with wheat [151].

Jalgaonkar et al. [149] evaluated quality changes in pearl-millet-flour-based pasta during six months of storage in biaxially oriented polypropylene packaging. The authors reported a consistent increase in quality parameters associated with deterioration: FFAs (0.48–0.82%), peroxide value (2.10–5.79 meq kg^−1^ oil), and lipid acidity (20.54–37.77 mg 100 g^−1^). In another study involving pasta made from pearl millet flour and semolina in varying proportions, Jalgaonkar et al. [150] reported that particle size influenced pasta composition and that a formulation containing 100% pearl millet flour was not viable because it disintegrated after cooking.

The results show that pearl millet is a versatile ingredient that can improve the nutritional and functional profile of various food matrices, even after grain processing and final product formation. However, proportions must be adjusted to avoid technological issues.

## 8. Sensory Acceptability of Pearl Millet Products in Non-Traditional Consumer Markets

Although pearl millet is predominantly cultivated in Brazil as a cover crop, forage and for seed production for animal feed [5], its historical trajectory as a food grain native to Africa and Asia reflects a profound ethnobotanical dimension. In the contemporary Brazilian context, efforts are underway to reframe pearl millet within the realm of human nutrition, shifting its perception from a mere forage input to a resilient, gluten-free functional ingredient.

However, sensory acceptance poses a critical barrier to this adoption in regions where it is not a traditional staple food. In Brazil, cereal-based food products have historically been evaluated with wheat flour serving as the sensory benchmark. Pearl millet flour differs from wheat flour, exhibiting a greenish-gray hue, earthy notes, and slight bitterness or astringency, characteristics linked to its phenolic and lipid profiles. From an ethnobotanical and cultural perspective, while traditional populations in Africa and Asia developed culinary practices that integrate these sensory attributes into daily staples, Brazilian dietary habits evolved predominantly around wheat, corn, and cassava. Consequently, local consumers may perceive pearl millet’s sensory attributes as unfamiliar to their palates. Bridging this gap requires understanding how traditional processing methods can be adapted to leverage consumer familiarity with conventional flours. The current literature relies largely on sensory evaluation data from populations with established pearl millet consumption habits; nevertheless, panels involving Brazilian participants already indicate the grain’s potential for human consumption [33,148,152,153,154]. Furthermore, panels with a small number of participants, especially trained ones, may not reflect the actual acceptability of the products, making larger panels with diverse members necessary. Table 9 presents products made with the addition of pearl millet and their respective sensory evaluations.

When pearl millet flour is used in gluten-free products, processing or combining it with other ingredients can enhance acceptance. Gluten-free breads formulated with maize starch and varying amounts of pearl millet flour and water (50% and 100%, 100% and 100%, 50% and 130%, 100% and 130%, and 75% and 115%) were evaluated by 52 regular bread consumers using a 10 cm hybrid scale. The parameters of appearance, color, and odor showed no statistically significant differences regarding the pearl millet flour and water proportions, with scores above 5.75. The formulations receiving the highest ratings were those with 50% flour/130% water and 75% flour/115% water, with overall scores of 7.83 and 7.73, respectively [152]. A similar acceptance result was reported by Silva et al. [153] when evaluating gluten-free breads made with rice flour (50%), potato starch (30%), and cassava starch (20%), with partial or total substitution by pearl millet flour at levels of 0%, 10%, 30%, 50%, or 100%. A total of 100 participants evaluated the breads based on overall acceptability on a 9-point hedonic scale. Formulations with 30% and 50% substitution received scores of 6.8 and 6.3, respectively, values close to that assigned to the commercial gluten-free bread (6.9).

In another study, the acceptability of a gluten-free pasta made from raw whole millet and precooked flours via thermoplastic extrusion (1:1) was evaluated by 86 untrained panelists using a 9-point hedonic scale and an acceptability index. Appearance received the highest score (6.1), tied with flavor (6.1). The overall assessment score was 5.9, while the lowest score was assigned to texture (5.2). The acceptability index varied across the evaluated parameters: 66% for overall acceptance, 68% for appearance and flavor, and 65% for texture [154].

Brasil et al. [148] evaluated the inclusion of hydrated or roasted pearl millet flour in a meat product (kibbeh) in two steps: first, 57 untrained participants assessed specific sample attributes using a 9-point hedonic scale and an acceptability index. Next, potential consumers (*n* = 92) evaluated sample acceptability at a supermarket. The authors reported no statistically significant differences among the samples in the acceptability index. Furthermore, all attributes received mean scores above six, indicating good acceptability compared to conventional preparations.

Cakes made with extruded pearl millet flour and supplemented with fiber (oat bran, rice bran, wheat bran, and psyllium) were evaluated by 104 untrained participants using a 9-point hedonic scale. The control formulation (without added fiber) achieved the best mean scores for color, aroma, flavor, and texture (7.88, 7.60, 7.61, and 7.83, respectively), followed by the formulation with psyllium (7.40, 7.36, 7.39, and 7.45, respectively). Overall acceptance remained above 7 for all formulations, except the one containing rice bran (6.59). Furthermore, purchase intention remained above 3.1 on a 5-point scale (ranging from 1, “definitely would not buy” to 5, “definitely would buy”), indicating good acceptability. The authors also reported that 60% of the participants were unfamiliar with pearl millet prior to the analysis, and 82% had never consumed products containing the cereal, suggesting market potential as well as an opportunity to add value to an underutilized grain [33].

In studies involving international panels of populations accustomed to consuming millet, sensory evaluation scores can vary depending on the ingredient blended with the product. Manchanda et al. [140] obtained scores above 7.6 on a 9-point hedonic scale (using 20 semi-trained panelists) for attributes such as appearance, color, taste, flavor, and texture in biscuits made from a blend of finger millet, pearl millet, and buckwheat flours (1.5:1:1), even after storage in polypropylene packaging at room temperature for 14 days. It was reported that taste and flavor decreased moderately over time (8.05, 8.00 and 7.89 and 8.00, 7.84 and 7.63, respectively), while texture remained unchanged during storage, with the mean value remaining constant (8.00). Overall acceptability decreased moderately at each interval, shifting from 8.263 to 8.158 and then to 8.053 after 14 days. This indicates that storage did not cause major or significant changes in the assessment of sensory properties. The same pattern was observed in cookies made with a blend of pearl millet and finger millet flour (1:1), which 30 untrained participants evaluated and rated above 4.5 (5-point scale) for texture, aroma, color, taste, and overall acceptability [141].

Kulkarni et al. [35] evaluated cookies prepared by replacing wheat flour with pearl millet flour at varying levels (20%, 40%, 60%, 80%, and 100%). Untrained panelists evaluated the cookies using a 9-point hedonic scale. Overall acceptability decreased significantly as the pearl millet flour content increased, dropping from 8.4 (20%) to 7.3 (100%) compared to the control sample containing only wheat flour (8.7). The increased amount of pearl millet flour imparted a gritty texture to the cookies, and panelists reported an undesirable grayish color, though the aroma of roasted millet was pleasant. Nevertheless, cookies formulated with up to 80% substitution maintained mean scores above 7.6 across all evaluated attributes. Hussain et al. [36] reported similar results when evaluating cookies made by substituting wheat with pearl millet flour washed with water or an alkaline solution (0.05 M NaOH) at various proportions (25%, 50%, 75%, and 100%). As the amount of pearl millet flour increased, overall acceptability decreased from 8 (100% wheat) to 3 (100% H_2_O-washed) and 4 (NaOH-washed). A bitter aftertaste was observed in the cookies with higher levels of substitution. Although washing the pearl millet flour with water or an alkaline solution mitigated this bitterness (which could be masked by higher sugar levels in the recipe), formulations with a substitution rate exceeding 50% showed lower acceptance.

Olatoye et al. [142] evaluated the acceptability of an instant beverage based on germinated, fermented, and milled pearl millet, supplemented with spices (ginger, pepper, and clove) and varying amounts of African locust bean pulp (ALBP). The beverages were evaluated by 50 untrained participants; the formulation without ALBP received the highest sensory scores on a 9-point scale (appearance 7.3, taste 6.4, aroma 7.2, mouthfeel 6.5, and overall acceptability 6.7). Scores decreased significantly as the amount of ALBP increased; however, none of the samples formulated were rejected. In another study by Atosona et al. [143], researchers prepared an instant beverage with a base composition similar to that of the previous study, fortified with baobab pulp (10, 15, and 20 g). Forty female adolescents, aged 10 to 19, evaluated the beverage using a 9-point hedonic scale. There were no significant differences regarding taste/sweetness, color, and flavor among the beverage samples with increasing levels of fortification, with mean scores above 7.2. The 10 g and 15 g fortification levels yielded the best overall acceptability (7.82 and 8.10, respectively) due to the balance of sweet and citrus notes. The authors further reported that a daily intake of 350 mL of the fortified beverage could meet up to 96% and 30.4% of the Recommended Daily Intake (RDI) of iron and vitamin C, respectively, among female adolescents.

Other studies have also evaluated the inclusion of pearl millet in milk-based formulations. In a beverage made from fermented pearl millet flour and milk, acceptability was assessed by 35 panelists; the highest score was given to color and appearance (7.11), followed by mouthfeel, taste, aroma, and flavor (6.65, 5.8, 5.8, and 6.5, respectively). Overall acceptability averaged 6.6 (“liked moderately”), and it is assumed that the pearl millet variety used had the greatest influence on consumer acceptability [144]. In another study, Joshi et al. [145] evaluated the acceptability of a probiotic strained yogurt-like dessert using a 9-point hedonic scale with 30 semi-trained panelists. The scores assigned to color and appearance ranged from 5.95 to 6.73, body and texture from 7.08 to 8.45, and overall acceptability from 6.67 to 8.01, and the shelf life was considered 28 days because counts decreased below the acceptable limit of 6 log10 CFU g^−1^ after this period. Patel et al. [146] evaluated the acceptability of a functional goat-milk-based nutritional supplement formulated with different concentrations of fermented finger millet and fermented pearl millet flours using an 8-point hedonic scale (where 8 indicated “very desirable” and 1 indicated “poor quality”) with a panel of 15 semi-trained panelists (students, research scholars, and scientists aged 22–45 years). Overall, scores were above five points, and the formulation containing 24% fermented finger millet and fermented pearl millet flours received the highest ratings, with values close to 7.

Yogurts prepared with malted pearl millet flour in ratios of 70:30, 60:40, and 50:50, evaluated by 13 panelists, received scores ranging from intermediate to low. Appearance, taste, and mouthfeel showed a downward trend as the amount of malted pearl millet flour increased (4.62, 4.46, and 4.23; 4.62, 4.08, and 4.08; and 4.8, 4.8, and 3.54, respectively). The lowest mean scores were assigned to aftertaste (3.15, 3.0, and 3.23), and overall acceptability remained around four points [147].

Jalgaonkar et al. [149] evaluated the sensory acceptability of pasta produced from a blend of pearl millet flour and semolina (50:50) over 180 days of storage, using a panel of 10 tasters and a 9-point hedonic scale. The authors reported no statistically significant differences in the evaluated attributes over time (appearance, texture, taste, and color), with mean scores remaining above 6.5, while overall acceptability remained above 7.50 throughout storage. In another study, 25 trained panelists evaluated waffles formulated with blends of wheat, finger millet, and pearl millet flours in proportions of 50:30:20, 50:25:25, and 40:40:20. The 50:30:20 and 40:40:20 formulations received overall quality scores of 68.5 and 66.5 (“very good”), respectively, while the 50:25:25 formulation achieved a mean score of 63.0 (“good”) [151].

Ultimately, sensory acceptance is dynamic and culturally mediated rather than static. As demonstrated, incorporating pearl millet into culinary preparations already established in the Brazilian diet yields satisfactory acceptance rates, even among consumers unfamiliar with the grain. Therefore, future research should combine ethnobotanical knowledge, leveraging regional food preparation habits and traditional processing techniques, with sensory testing involving broader consumer panels. This integrated approach will be crucial for optimizing formulations that maximize both nutritional density and market suitability in regions where pearl millet consumption is not customary.

## 9. Market Barriers for Pearl Millet Flour and Ready-to-Eat Products in Brazil

The barriers examined in the previous sections do not operate independently in a commercial context. A flour must simultaneously gain acceptance from consumers unfamiliar with the grain (Section 8), withstand distribution, preserve the nutrients that justify its use, and behave predictably in formulations developed for wheat. The three constraints discussed below are presented in the order in which they arise along the supply chain and are interdependent: an intervention that resolves one often exacerbates another. Furthermore, the integrated analysis provides an overview of the points previously discussed.

### 9.1. Deterioration and Rancidity of Pearl Millet Flour

For pearl millet, shelf life is not merely one commercial attribute among others; it is a condition for market entry. Under ambient conditions, non-stabilized flour becomes sensorially unacceptable well within the turnover period of a conventional retail chain due to lipase-mediated hydrolysis and lipoxygenase-mediated oxidation of the lipid fraction (Section 6). In a country of continental dimensions, where flour may travel long distances under elevated ambient temperatures and be stored without refrigeration at the point of sale, a product with a two- to three-week window is not commercially distributable, regardless of its nutritional merit. Stabilization is therefore not optional. The relevant question for the Brazilian market is not whether shelf life can be extended under laboratory conditions, but which stabilization routes remain effective under tropical temperature and relative humidity, and at what processing cost. To our knowledge, accelerated shelf-life studies conducted under conditions representative of Brazilian distribution are nonexistent.

The deterioration of the sensory characteristics during storage is predominantly associated with lipid oxidation, which occurs mainly on unsaturated fatty acids, such as linoleic (ω-6) and α-linolenic (ω-3) acids [155].

The parameters indicating rancidification are the acidity index (hydrolytic) and the peroxide value (oxidative), mediated by the enzymes lipase and lipoxygenase (LOX). Hydrolytic rancidity (mediated by lipase) occurs when fats are broken down by water or by the enzymatic degradation of lipids into FFAs (cleavage of triacylglycerols), while oxidative rancidity (mediated by LOX) occurs when unsaturated fats react with atmospheric oxygen through chemical oxidation or enzymatic degradation of polyunsaturated fatty acids into hydroperoxides, aldehydes, and volatile compounds. This process generates off-flavors in flour and is the main cause of rancidity development, accelerated deterioration, and reduced shelf life during storage [29,156,157,158].

The expression and activity of these mechanisms can be under genetic control, involving both nuclear inheritance components and factors of cytoplasmic origin [159]. Nuclear–cytoplasmic interaction, the interaction between the nuclear and cytoplasmic genomes, can play a decisive role in modulating the biochemical processes associated with rancidity development in pearl millet flour. Identifying and exploiting favorable combinations between these genomes can help develop hybrids with superior grain quality attributes, greater stability during storage, and extended shelf life. This approach addresses the main challenges of rancidity and flour stability during storage, as well as pearl millet’s potential to meet food security challenges across various regions [160].

A study evaluated which cytoplasmic profile significantly influences the activity of enzymes associated with rancidification in pearl millet, identifying the cytoplasm type that supports a higher rate of lipid degradation. The research demonstrated the importance of considering nuclear–cytoplasmic interactions and genotypic constitution in breeding programs aimed at selecting combinations more favorable to reducing rancidity and improving post-processing stability of pearl millet grains [159]. These advances can improve consumer acceptance, expand market potential, and add value to pearl millet as a raw material for the food industry.

Furthermore, traditional processing techniques, such as decorticating and extruding (explored in Section 6), are widely used and can effectively reduce enzymatic activity. However, other technologies can achieve similar results and help identify genotypes less susceptible to deterioration. Thermal processing combined with efficient, nitrogen-flushed laminated packaging inactivates lipolytic enzymes, preventing the development of undesirable flavors and extending the shelf life of flour and processed food products by approximately 60 days [161]. Airtight storage is also reported to limit oxygen availability, thereby retarding the oxidation of unsaturated fatty acids, such as oleic and linolenic acids, that are prone to deterioration. Furthermore, the technique inhibits lipase enzyme activity, reducing triglyceride breakdown and the risk of rancidity [162].

Metabolic profiling is a grain research strategy that characterizes quality attributes, nutritional composition, and responses to biotic and abiotic stresses by elucidating interactions among genotype, environment, and phenotype [163]. Metabolomic analysis is an advanced omics approach that comprehensively characterizes the set of small-molecule metabolites (metabolome) present in biological systems [164]. This approach employs sophisticated analytical platforms, such as gas or liquid chromatography coupled with mass spectrometry (GC-MS, LC-MS) and nuclear magnetic resonance spectroscopy (NMR). A study using this methodology identified 25 discriminatory metabolites associated with the progression of rancidity and oxidative stress, including phytol, ethanolamine, chlorophyllide b, and glycoside derivatives, that show potential as rancidity biomarkers. These markers can support the development of metabolic sensors and technological strategies to enhance the stability and expand the application of pearl millet flour in processed foods [165].

Metabolomics offers a new perspective for elucidating biochemical processes and enabling precision genetic improvement, with the aim of developing pearl millet lines with greater stability and lower susceptibility to rancidity. A study employing untargeted metabolomics via high-resolution mass spectrometry (LC-HRMS) characterized the metabolic profiles of ten pearl millet genotypes subjected to accelerated aging, identifying metabolites primarily associated with fatty acid, glycerophospholipid, sphingolipid, flavonoid, and terpenoid pathways. More susceptible genotypes showed higher abundances of aldehydes, alcohols, esters, fatty acyls, and fatty amides. Quantification of stearic, palmitic, linoleic, and linolenic acids confirmed the link between altered lipid metabolism and rancidity development, highlighting metabolomics’ potential to select more stable genotypes [166].

Analysis of the phospholipase gene family (enzymes that catalyze phospholipid hydrolysis in cell signaling and stress responses) identified 44 genes in pearl millet, five of which showed preferential expression in the grain, suggesting direct involvement in regulating acidity [167].

Thus, it is crucial to identify biochemical indicators, such as peroxide and acidity levels, and to understand enzyme activities (lipase, LOX, and peroxidases), processing and storage dynamics, and genotypic diversity; this enables the identification of specific genotypes that are less prone to lipid degradation. Furthermore, expanding the metabolomics approach with NMR and LC-HRMS enables advanced tools to improve pearl millet cultivars and enhance oxidative stability [165,168].

### 9.2. Nutrient Gains and Losses Across Processing

The processes that stabilize the flour largely overlap with those that reduce its nutritional value (Section 6). Thermal and thermomechanical treatments inactivate lipolytic enzymes but also affect phenolic compounds, fibers, and heat-sensitive micronutrients; decorticating reduces both antinutrients and the mineral-rich fractions that contain them. The nutritionally relevant metric is therefore neither the composition of the grain nor that of the freshly milled flour, but rather the bioaccessible nutrient content, a value that has rarely been reported. This gap has direct market consequences: nutritional claims are based on the composition of the raw material, whereas consumers purchase the stabilized and stored product. Establishing the compositional trajectory throughout processing and storage, expressed in bioaccessible rather than total terms, is a prerequisite for any evidence-based claim regarding pearl millet flour.

Pearl millet is a cereal known for its nutritional and functional potential in Asia and Africa; however, composition is not the only factor that reaches the consumer. The relevant question for commercial flour is not only what the grain contains but also what survives after processing, storage, and digestion. Therefore, it is important to differentiate three micronutrient measures: nutrient content is the amount quantified in the grain or flour; bioaccessibility is the fraction released from the food matrix during digestion; and bioavailability is the fraction absorbed and used by the body [169,170,171]. A high content of iron, zinc, phenolics, or fiber therefore does not by itself indicate nutritional value at the physiological level. Although pearl millet contains high concentrations of iron and zinc, its grain is surrounded by a tough fibrous seed coat rich in antinutritional factors, such as phytic acid and polyphenols, which, in high quantities, can form complexes with dietary minerals including calcium, zinc, and iron, resulting in a significant reduction in their bioaccessibility and bioavailability [169,171].

Even before processing, the fraction of iron and zinc released from the matrix under simulated digestion is low across thirteen cultivars; bioaccessibility reached at most about 18% for iron and 15% for zinc, and only in genotypes with low phytic acid [169]. Minerals and antinutritional factors occupy the same anatomical tissues of the pearl millet grain. The physical removal required by milling and decortication, common techniques used for millet grain, revealed the basis for the effects of processing on the bioavailability of minerals in pearl millet as described by Krishnan and Meera [172], who demonstrated that sequential milling of two cultivars showed increased iron content in the bran fractions with decortication time, while polyphenols and phytic acid, initially low, increased in parallel. Moreover, microscopic analysis confirmed colocalization of micronutrients and inhibitory factors within the same tissues; removing approximately 10–15% of the bran resulted in the highest bioavailability of iron and zinc [172].

Suma and Urooj [107] investigated how dry milling redistributes nutrients, antinutrients, and mineral bioavailability across pearl millet flour fractions. They processed two cultivars, Kalukombu and Maharashtra Rabi Bajra, into three flour types: whole, bran-rich, and semi-refined. The bran-rich fraction retained the highest ash content (3.3 g 100 g^−1^ in Kalukombu and 3.5 g 100 g^−1^ in Maharashtra Rabi Bajra) and contained higher concentrations of phosphorus, calcium, and antinutrients such as oxalate and phytate. Except for fat, which was only 1.3%, the semi-refined flour was compositionally close to the whole flour, with the lipids, minerals, and antinutrients presumably having been retained in the coarse, bran-rich fraction. The study found that total iron was significantly lower in the semi-refined and bran-rich fractions than in whole flour, yet iron bioaccessibility was highest in the semi-refined flour, intermediate in the whole flour, and lowest in the bran-rich fraction. Whole flour bioaccessibility was lower in absolute terms, at approximately 3% for Kalukombu and 7% for Maharashtra Rabi Bajra. Although semi-refined flour contained fewer minerals than whole flour, it had lower antinutrient content [107]. These findings show that pearl millet flour has no fixed nutritional identity; its behavior depends on the processing route, which redistributes nutrients, antinutrients, and lipids across fractions rather than removing them, so each route trades one nutritional attribute for another.

In addition to physical processing, thermal treatments such as hydrothermal, microwave, and infrared treatments on pearl millet roti, a flatbread traditionally used in India, and the evaluation of these treatments over three stages in an in vitro digestion model (oral, gastric, and intestinal), were observed in the study by Ali et al. [173], revealing changes in the bioavailability of iron, zinc, and phenolic compounds depending on the treatment method. Hydrothermal treatment lowered the inherent glycemic potential from 72.4% to 60.4% and reduced rancidity-associated enzyme activity in the flour. Moreover, increased starch structural order reduced the bioaccessibility of phenolics, iron, and zinc, whereas microwave and infrared treatments elicited distinct matrix responses [173].

Fermentation is another processing method used for pearl millet for food consumption. In millet injera and millet-maize-based injera, a fermented flatbread, prolonged fermentation reduced the phytate and tannin content and increased the bioavailability of minerals such as iron, zinc, and calcium; these parameters improved in samples prepared only with pearl millet. In contrast, fermentation time decreased fiber content, attributed to the partial solubilization of cellulose and hemicellulose by microbial enzymes. Thus, these findings contradict the idea that processing losses are entirely prohibitive, as they also increase the bioavailability of other nutrient classes and bioactive substances [174].

It is important to emphasize that all the articles addressed the mineral bioaccessibility in vitro; however, a recent study using a Brazilian cultivar demonstrated mineral bioavailability in vivo. The study demonstrated that whole-grain pre-cooked pearl millet flours classified as conventional, germinated, and extrusion-precooked restored blood iron concentration in iron-depleted Wistar rats and improved inflammation and oxidative stress markers, with germinated flour outperforming extruded flour in iron metabolism [101]. Moreover, the three processed flours, mainly germination, increased microbial diversity and improved intestinal morphology [175].

Bioaccessibility and, more recently, bioavailability data provide the same conclusion: pearl millet displays nutritional value. However, this conclusion was reached using freshly processed flour and rodent models, and published studies on clinical trials in humans are limited. Nor does this evidence address what lies between nutritional value and consumption: a flour that must survive tropical distribution, and food choices in which nutritional attributes compete with habit, familiarity, price, and culinary convention. The available evidence supports a place for pearl millet in the Brazilian diet. Confirming its benefits in humans and building consumer familiarity with the grain are the next steps, and both extend beyond what compositional and preclinical data can establish.

### 9.3. Technological Handling of Gluten-Free Flour and the Dilemma of Flour Blends

The absence of gluten eliminates the viscoelastic network responsible for gas retention, dough handling, and crumb structure. Consequently, pearl millet flour used in isolation yields products with low specific volume, dense crumb, and accelerated stalling, requiring compensation through processing or ingredient additions to achieve adequate performance. In domestic and small-scale production in Brazil, the practical response has been the use of composite flours, either as a partial replacement for wheat flour or in combination with other flours (Section 7). This solution creates a commercial contradiction that warrants clarification. While the blend restores workability and consumer acceptance, it dilutes the fiber, mineral, and phenolic compound content that drives the grain’s use; furthermore, when blended with wheat, it completely eliminates the “gluten-free” status, the attribute commanding the highest market value and, under Brazilian labeling regulations, a claim incompatible with any formulation containing wheat [176]. The open question is quantitative: what substitution range allows the product to retain its characteristics while still qualifying for the claims that justify its price positioning?

To replicate the viscoelastic properties of wheat flour dough, gluten-free (GF) products are formulated with complex blends of gluten-free flours to maximize similarity to conventional products and reduce consumer resistance to the developed items. Furthermore, the literature reports extrusion and germination as strategies for producing GF products [177].

Pessanha et al. [138] evaluated the technological quality of GF breads made with raw and extruded pearl millet flour, used either alone or in a 50:50 blend. Samples made with the individual flours exhibited the highest hardness values (extruded: 3957 N; raw: 922 N). However, when blended, hardness decreased to 623 N and bread volume increased to 0.93 cm^3^ g^−1^. Despite this reduction, the flour combination significantly increased cohesiveness (0.534), resulting in the highest chewiness value (900 N) among all formulations. This behavior reflects a rubberier crumb structure that resists oral disintegration.

In another study, Marinho et al. [139] prepared breads using the straight-dough and sponge-dough methods, incorporating blends of raw and extruded pearl millet flour (50:50) and raw, extruded, and germinated pearl millet flour (45.5:45.5). The breads exhibited high hardness; the sponge-and-dough method yielded higher values (15.51–20.25 N) compared to the straight-dough method (12.59–12.62 N), a result that may be linked to the increased dietary fiber content. The authors also noted that adding germinated flour resulted in greater softness and improved recovery capacity after removing applied stress.

In products made from a blend of wheat flour and pearl millet flour, the properties of the mixture also undergo changes. Costa et al. [33] observed this in cakes prepared with extruded pearl millet flour and wheat flour, supplemented with various fiber sources (oat bran, rice bran, wheat bran, and psyllium). The specific volume of the cakes ranged from 1.74 to 1.91 cm^3^ g^−1^; however, no statistical difference was observed. Regarding the texture profile, the control cake (without added fiber) exhibited the lowest firmness (6.62 N) and the highest springiness (0.72) and cohesiveness (0.36), indicating a product that does not disintegrate easily. The control formulation and the formulation with added psyllium minimized undesirable characteristics such as crumbling and grittiness.

Composite flours made from wheat, pearl millet, and orange peel at various concentrations were evaluated for biscuit production. Composite flours varied significantly with substitution level. An increase in water absorption capacity was observed, rising from 0.10 g g^−1^ in the control formulation (100% wheat flour) to 1.15 g g^−1^ in the formulation containing 60% wheat flour, 32% pearl millet flour, and 8% orange peel flour, due to the higher fiber content, making these composite flours advantageous for use in bakery products. Oil retention capacity also increased (ranging from 1.53 to 1.90 g g^−1^), making them ideal for baked goods where fat retention improves palatability and flavor retention. However, as the concentrations of pearl millet flour and orange peel flour increased, the hardness of the biscuits rose from 7.53 N to 8.75 N, owing to the presence of proteins and fibers [34].

Kulkarni, Sakhale, and Chavan [35] evaluated the technological properties of low-gluten cookies made from a blend of pearl millet and wheat flours at various concentrations. Consistent with other reported studies, as the substitution of wheat with pearl millet flour increased, firmness rose from 19.24 N (100% wheat flour) to 28.91 N (100% pearl millet flour). Furthermore, values for chewiness (10.21–13.68 N), gumminess (12.47–24.42 N), breaking strength (28.78–43.00 N), and cutting strength (31.39–44.20 N) also increased with higher levels of pearl millet flour, resulting in more compact and firm cookies. However, Hussain et al. [36] showed that grain processing can positively influence cookie hardness and fracturability. Washing the grains for 24 h in distilled water or an alkaline solution (0.05 M NaOH) made it possible to significantly reduce cookie hardness from 5798 g (100% wheat flour) to 4323 g (100% H_2_O-washed) and 4053 g (100% NaOH-washed), and fracturability from 8.37 mm to 6.7 mm and 6.68 mm, respectively, possibly due to the dilution of the gluten protein.

Regarding pasta products, Jalgaonkar and Jha [150] evaluated formulations with varying proportions of semolina flour and pearl millet flour. As the pearl millet flour content increased, the pasta exhibited higher cooking loss, rising from 5.39% (100% wheat flour) to 8.70% (100% pearl millet flour). Furthermore, decreases were reported in hardness (20.81–7.48 N), chewiness (14.52–2.30 N), gumminess (14.09–3.87 N), springiness (1.03–0.59 mm), and cohesiveness (0.68–0.52), attributed to the weakening of the protein network, gluten content, and starch leaching. In another study by Jalgaonkar et al. [149], pasta made from a blend of semolina and pearl millet flour (50:50) was evaluated during storage for up to 180 days; cooking loss increased from 6.22% (0 days) to 7.66% (180 days). However, hardness, cohesiveness, springiness, gumminess, and chewiness remained stable over time.

Collectively, these findings highlight the technological impact of incorporating pearl millet, whether raw, processed, or in blends, into bakery and pasta matrices. Although the grain significantly enhances nutritional value, its inclusion systematically alters textural parameters, increasing hardness and chewiness in baked goods while compromising structural integrity in pasta formulations. Overcoming the challenges associated with composite flours requires adapting processing techniques and optimizing substitution ratios. Balancing these physicochemical interactions is essential to maximizing the grain’s dietary benefits without compromising texture, volume, or consumer acceptance.

### 9.4. Contaminants in Pearl Millet Flour

Pearl millet is susceptible to contamination by various agents, which represents an important and serious threat to crop yield and, above all, to the quality of millet-derived products, particularly flour. The potential contamination of pearl millet flour by different factors is concerning and can negatively impact food safety, human health, and the global agricultural landscape. In this context, a multidisciplinary approach is essential, encompassing fields such as microbiology, agriculture, food science, and regulatory compliance [37].

Pearl millet flour harbors a diverse native microbiome that can proliferate rapidly under inadequate storage conditions. The total bacterial and fungal counts can increase significantly throughout storage, especially at temperatures above 25 °C. Contamination by potentially mycotoxigenic fungi and the consequent production of mycotoxins can compromise the quality of grains and processed products, as well as pose risks to trade and food safety. Among the main fungal genera isolated from pearl millet are *Fusarium*, *Aspergillus*, *Penicillium*, *Curvularia*, *Alternaria*, *Rhizopus*, and *Mucor* [178]. Mycotoxins constitute a major chemical hazard to the safety of pearl millet flour, particularly during storage and processing. Aflatoxins, produced mainly by *Aspergillus flavus* and *Aspergillus parasiticus*, also represent a relevant concern in pearl millet-derived products. It is worth noting that storage at sub-zero temperatures, such as −5 °C, can halt fungal growth and contribute to product preservation [179].

Although bacterial loads may remain relatively stable under low-moisture conditions, inadequate processing and increased moisture content can favor rapid microbial proliferation and accelerate flour deterioration [180]. Contamination can also be associated with the type and characteristics of the packaging material used, as its protective capacity against moisture, oxygen, and external contaminants directly influences product stability during storage [181]. Furthermore, storage pests constitute another major hazard to pearl millet flour. During milling, the removal of the grain’s protective outer structures increases the product’s exposure and can favor insect infestation, compromising both the quality and safety of the flour. Thus, the microbiological quality and safety of pearl millet flour must be considered in an integrated manner from grain production and harvesting to processing, packaging, and storage. Controlling moisture, temperature, processing conditions, packaging characteristics, and storage pests is essential to reduce deterioration and limit the occurrence of fungi and mycotoxins. These factors reinforce that the use of pearl millet as a raw material for human consumption depends not only on its nutritional composition and technological functionality, but also on the implementation of hazard control strategies throughout the entire production chain [180]. Figure 2 summarizes the pathway of pearl millet from cultivation to human consumption in Brazil and the barriers along it.

## 10. Integrated Analysis: From Field to Consumer

The starting point for an integrated analysis from field to consumer is the characteristics of the grain, whose lipid profile, minerals, fibers, and phenolic compounds confer nutritional potential while also creating technological challenges. The high lipid content favors flour deterioration after milling due to increased lipid exposure, with risks of rancidity and alterations in aroma and flavor. Thus, a relevant nutritional characteristic in the field turns into a post-harvest stability limitation [157].

The main conflict in the supply chain lies in processing, as techniques such as decortication, milling, thermal treatments, and extrusion can reduce enzymatic activity and extend shelf life, but they also modify the food matrix. In decortication, for example, antinutritional factors are removed simultaneously with fractions where minerals are concentrated. However, the removal of phytates and polyphenols increases the bioaccessibility of iron and zinc [170]. Therefore, from a commercial and nutritional perspective, the initial grain composition is less critical than the final nutrient bioavailability for absorption. This distinction among chemical composition, bioaccessibility, and bioavailability is fundamental, as different processing routes redistribute lipids, minerals, fibers, and antinutrients, producing nutritionally distinct flours. Furthermore, fermentation and germination exemplify processes in which an apparent compositional “loss” is compensated for by greater nutrient availability or favorable matrix modifications [171].

There is also an important connection between genotype and processing. Susceptibility to rancidity is not necessarily uniform across cultivars. Metabolomics approaches, biochemical markers, and gene expression studies can enable the selection of naturally more stable genotypes. This shifts part of the technological solution back to the field itself, as instead of relying exclusively on post-harvest treatments, it becomes possible to develop raw materials that are genetically less susceptible to deterioration [166].

Another critical challenge is observed during storage due to the rapid development of rancidity before reaching the consumer. Therefore, thermal stabilization, low-oxygen packaging, and the control of storage conditions are essential strategies [157]. However, these technologies must be evaluated under conditions representative of the Brazilian scenario, particularly given the elevated temperature and humidity. In this context, a gap in the literature is identified regarding accelerated shelf-life studies that accurately simulate the tropical distribution chain in Brazil.

In the subsequent step, product formulation challenges arise regarding dough structure due to the gluten-free nature of the grain. Blending with other flours can improve texture, processing, and sensory acceptance; however, it proportionally reduces the concentrations of minerals, fibers, and bioactive compounds [182]. Thus, the challenge arises to determine the ideal level of pearl millet flour incorporation, ensuring technological functionality and sensory acceptance without excessively diluting its nutritional benefits.

Finally, closing the loop, the acceptance by Brazilian consumers comes into focus, as it will depend heavily on product formulation, processing, and the proportion of pearl millet used, rather than simply on the intrinsic quality of the grain. Products with higher millet concentrations may face significant technological and sensory barriers; thus, factors such as sensory perception, familiarity, dietary habits, price, and convenience ultimately determine market acceptance and consumption [17].

An integrated analysis demonstrates that the steps of the pearl millet value chain—field, processing, storage, formulation, and consumption—operate as an interconnected system of technological trade-offs. For instance, improving stability may result in a reduction in certain nutrients; preserving these nutrients can complicate processing stages; and enhancing sensory acceptance may require blending pearl millet with other grains. Undoubtedly, each stage alters the quality that ultimately reaches the consumer. This integrated perspective highlights why priority research gaps include shelf-life stability studies under Brazilian environmental conditions, post-processing and storage bioaccessibility evaluations, human clinical trials, and sensory testing with local consumers.

Regarding the transition of pearl millet from a cover crop to a food crop, the occurrence of pearl millet in Brazilian agricultural systems, particularly as a cover crop, does not imply that the grain produced in these systems is automatically intended or suitable for human consumption. This transition requires a clear distinction between the agronomic objectives and quality criteria associated with each production system. Pearl millet production aimed at biomass generation establishes selection and management criteria predominantly related to biomass yield, soil cover, nutrient cycling, and adaptability to the farming system. Conversely, for the intentional production of grains for human consumption, beyond agronomic performance and ecosystem services, factors such as grain yield and quality, nutritional composition, crop health, food safety, ease of harvesting, post-harvest stability, and technological processing suitability must be considered [17].

In this context, the importance of genotype-by-environment (*G* × *E*) interaction becomes clear, as the adaptation of a cultivar for biomass production or to a specific region does not guarantee consistent performance or quality when the goal shifts to food grain production. Environmental conditions during grain filling and maturation can alter key agronomic traits and compositional attributes relevant to food applications. Therefore, identifying suitable genetic material requires dedicated evaluations focused not only on yield, but also on grain quality and stability. Furthermore, incorporating grains harvested from cover crop systems into the food supply chain depends on harvesting and post-harvest management practices that are fully compatible with food-grade standards [17].

Furthermore, incorporating grains harvested from systems originally intended for cover cropping into the food supply chain depends on appropriate harvesting, drying, cleaning, storage, and transport practices, as well as the control of contaminants, residues, and deterioration, along with the maintenance of raw material traceability and segregation. Consequently, the food use of pearl millet produced in cover crop systems should not be defined merely by crop origin or identity, but by proof that the genotype (supported by plant breeding studies), environmental conditions, and production and post-harvest practices fulfill the quality and safety requirements applicable to food products [183]. This framework expands the concept of “cover crop to food crop” from a simple shift in target purpose to an integrated production system transition, in which grain quality must be safeguarded from genetic material selection through to its entry into the food chain.

## 11. Conclusions and Future Perspective

Although pearl millet is not commonly consumed outside Asia and Africa, it offers a diverse nutritional profile that can contribute to food and nutritional security. This review presented a critical point: pearl millet is grown on a large scale in Brazil, and it is almost never consumed as food by the population. The evidence in this review suggests the reason may be primarily related to the lack of knowledge regarding the crop and its market, making it necessary to disseminate knowledge about its cultivation and stimulate the development of food products.

The challenges that emerge between the field and the plate are interconnected. The lipid content and profile that distinguish pearl millet nutritionally also shorten the shelf life of its flour, and the processing technologies used to prevent deterioration affect the same fractions that carry minerals, phenolic compounds, and fiber. Because pearl millet is a gluten-free grain, handling its dough is difficult; while mixing it with other flours can restore workability, it may diminish the nutritional properties that justify its use. Consumers ultimately judge all of this by comparing cereal-based foods with references they already know. Established dietary habits, in Brazil as elsewhere, leave little familiarity for a grain such as pearl millet, which enters the market without a culinary tradition to support it.

Four priorities emerge for future research. Accelerated shelf-life studies should be conducted with kinetic extrapolation anchored to the temperature and humidity encountered in Brazilian distribution, rather than to the temperate conditions assumed in most of the literature. Nutrient bioaccessibility should be measured in stabilized flour after defined storage periods, since this is the product that consumers purchase. Human clinical trials are needed since in vivo evidence comes from animal models. Finally, sensory studies should be conducted with panels representative of the target market and across different storage times to identify the proportion of pearl millet flour in a blend at which acceptance and nutritional benefit coexist. The current scientific literature on pearl millet is abundant but geographically concentrated, as the grain is well characterized in Africa and Asia but remains understudied and underconsumed elsewhere. Closing that gap depends less on further compositional description and more on processing, consumer research, and evidence generated under Brazilian conditions.

Grain quality and performance depend on the interaction among grain characteristics, processing and storage steps, product functionality, and consumer acceptance, while also being influenced by the food system’s infrastructure. Thus, an integrated approach encompassing these factors is essential to ensure higher quality, safety, and market acceptance of the products.

## Figures and Tables

**Table 1:** Nutritional composition of pearl millet and other cereals.

Proximate Composition (g 100 g^−1^)	Moisture	Carbohydrates	Dietary Fiber	Proteins	Lipids	Ash	Ref.
Pearl millet, flour	8.73–13.49 (11.17)	66.57–69.87 (68.07)	11.90–13.30 (12.60)	8.00–10.98 (9.33)	6.10–8.16 (7.04)	0.73–1.65 (1.33)	[7,9,10,19]
* Foxtail millet, grain	9.47–12.27 (10.64)	70.50–79.75 (76.11)	7.10–7.70 (7.40)	10.67–13.40 (11.78)	4.03–6.20 (5.18)	1.29–1.60 (1.44)	[38,39]
Refined wheat flour	11.10–11.34 (11.22)	74.27–74.60 (74.43)	2.76–3.00 (2.88)	10.36–12.0 (11.18)	0.76–1.70 (1.23)	0.51–0.56 (0.53)	[11,18]
Rice, raw	9.93–11.20 (10.56)	78.24–80.30 (79.27)	2.77–2.81 (2.79)	7.04–7.94 (7.49)	0.52–1.03 (0.77)	0.42–0.56 (0.49)	[11,18]
* Oats, grain	9.10–12.60 (10.23)	67.36–73.10 (70.84)	10.60	12.48–13.48 (13.03)	3.24–5.40 (4.13)	1.52–2.26 (1.77)	[40,41]

* Values are expressed on a dry-weight basis. The ranges (min.–max.) and midpoints reflect the natural compositional variability reported in selected, independent analytical studies.

**Table 6:** Metabolic and functional effects of pearl millet.

Processing	In Vivo			
	Model	Method	Effects	Ref.
Powdered pearl millet and ethanolic extract	48 adult male Wistar rats	Hyperlipidic diets with added powdered pearl millet and ethanolic extract. Duration of 8 weeks	Reduction in final body weight, liver, visceral fat deposit, and epididymal chemical variables. Dose-dependent relation	[98]
Germinated pearl millet flour	30 male Wistar rats aged 21 days	Hyperlipidic and hyper-fructose diet with added germinated pearl millet flour. Duration of 10 weeks	Reduction in adiposity and biochemical variables	[99]
Milled pearl millet	25 male mice aged between 10 and 20 weeks	Standard laboratory diet (4RF21^®^) supplemented with milled pearl millet. Duration of 4 weeks	Hypoglycemic, antioxidant, and anti-inflammatory effects	[100]
Pre-cooked conventional, germinated, and extruded whole pearl millet flours	40 Wistar rats aged 21 days	Diet AIN-93G without iron for 28 days; AIN-93G with added ferrous sulfate; AIN-93G and conventional pearl millet flour; AIN-93G and germinated pearl millet flour; and AIN-93G and extruded pearl millet flour for 21 days	Promising bioavailability of iron	[101]
Pearl millet starch-germ complex and germ by dry heat treatment (PMSGH)	25 participants, 12 healthy and 13 pre-diabetics	Formulation of control bread and bread with PMSGH in the proportions 20, 25, and 30%	Reduction in blood glucose levels and glycemic index in healthy and pre-diabetic individuals	[102]
	**In vitro**			
Pearl millet extract of phenolic compounds	Osteosarcoma U2OS cells	Extracts with concentrations of 20 to 80 µg mL^−1^	Stopping of cell cycle and apoptosis	[103]
Pearl millet ethanolic extracts	Inhibitory activity in lipase, α-amylase and α-glucosidase	Ethanolic extracts from 3 varieties of pearl millet at concentrations of 50, 100, 200, 400, 600, 800 and 1000 µg mL^−1^	Inhibitory activity of lipase (76.02%) and significant inhibition of amylase (82.88%) and glucosidase (82.83%)	[104]

**Table 7:** Main processing methods of pearl millet.

Processing	Principal Effects	Ref.
Physical methods		
Decortication	Reduction in lipoxygenase activity and lipid oxidation; Reduction in antinutrients; Reduction in lipids and in dietary fiber content; Reduction in bioactive substances; Reduction in antioxidant activity; Loss of essential micronutrients.	[22,105,106,117]
Milling	Reduction in antinutrients; Reduction in bioactive substances; Reduction in antioxidant activity; Reduction in dietary fiber content.	[22,107]
Physicochemical methods		
Soaking	Reduction in antinutrients; Increase in mineral bioavailability; Increase in free iron content; Nutrient leaching.	[22,108,109]
Acid treatment	Increase in iron bioavailability; Reduction in mineral content; Reduction in polyphenols; Reduction in fatty acid content.	[110,111]
Thermomechanical processes		
Roasting	Reduction in antinutrients; Reduction in phenolic compounds; Nutrient loss; Reduction in dietary fiber; Reduction in antioxidant activity.	[8,22,112]
Parboiling	Increase in phenolic compounds; Increase in antioxidant activity; Increase in resistant starch content; Reduction in glycemic index; Reduction in protein digestibility.	[22,113,120]
Cooking	Reduction in antinutrients; Reduction in bioactive substances; Reduction in antioxidant activity.	[22,114,115,116]
Extrusion	Reduction in antinutrients; Inhibition of lipase and lipoxygenase; Formation of amylose-lipid complexes; Slower in vitro digestion; Reduction in lipid content.	[117,118,119]
Biochemical processes		
Sprouting/Germination	Improvement in digestibility; Increase in bioavailability and bioaccessibility; Reduction in antinutrients; Reduction in enzymatic activity; Increase in protein, dietary fiber and carbohydrate content; Increase in magnesium, calcium and sodium content; Increase in phenolic compounds; Increase in antioxidant activity; Reduction in lipid content; Reduction of potassium and iron content.	[117,121,122,123]
Fermentation	Increase in bioactive substances; Reduction in antinutrients; Improvement of antioxidant potential; Improvement of DNA damage protection activity; Reduction in dietary fibers and mineral content; Increase in the production of the enzymes α-amylase, β-glucosidase and xylanase.	[22,124,125]

**Table 8:** Products elaborated from processed pearl millet grains.

Product	Processing	Formulation	Main Conclusions	Ref.
		Gluten-free		
Bread	Raw whole pearl millet flour and pearl millet flour through thermoplastic extrusion	Pure flour or mixed flour 50:50 (100%), water (35%), liquid eggs (35%), fat (8%), fine grain white sugar (10%), dry yeast (5%), salt (2%)	Extrusion increased antioxidant capacity, antihyperglycemic activity, and specific volume, while reducing the hardness of the bread obtained from the blended flour	[138]
	Raw millet flour (RMF), extruded millet flour (EMF) and germinated millet flour (GMF)	Six different flour bases (100%): RMF, EMF, RMF + EMF (50:50), RMF + EMF + GMF (47.5:47.5:5), RMF + GMF (95:5) and EMF + GMF (95:5), eggs (35%), sugar (6%), fat (6%), yeast (3%) and salt (2%)	Increased ash, dietary fiber, and phenolic content, with enhanced antioxidant activity and α-amylase inhibition	[139]
Biscuit	Pearl millet flour	Mix flour of finger millet, pearl millet and buckwheat 1.5:1:1 (75 g), sodium bicarbonate (0.5 g), calcium carbonate (0.2246 g), demerara sugar (30 g), butter (40 g) and beetroot extract (5–10 mL)	Higher protein content, enriched mineral profile, and high energy density	[140]
Cookies	Pearl millet flour	Mix flour of pearl millet and finger millet 1:1 (49%), butter (20%), sugar (20%), water (10%), and baking powder (1%)	Increased total phenolics, decreased glycemic index, and reduced sugar release	[141]
Instant beverage	Germinated and milled pearl millet grains; freeze-dried pearl millet sourdough	Pearl millet sourdough (200 g), ginger (2 g), pepper (2 g), clove (2 g) and water (2:1 w/v)	Presence of vitamins A and C, minerals, phenolic compounds, and flavonoids; increase in antioxidant activity	[142]
	Pre-hydrated, dried, and ground pearl millet powder	First mix–powder: pearl millet, ginger (50 g), chili pepper (4 g), cloves (4 g); second mix: millet powder and baobab fruit pulp (100:0, 100:10, 100:15, and 100:20), water (500 mL) and sugar (13.3 g)	Elevated mineral content, presence of citric acid and vitamin C, low tannin levels, and 9-month shelf life	[143]
Fermented beverage	Pearl millet fermented flour	Fermentation of pearl millet flour (10 g), skim milk (12 g), and water (100 mL) inoculated with *L. fermentum* MS005, followed by formulation of the fermented base with buttermilk (40%), black salt (0.7%), and spices (0.06% black pepper and 0.3% cumin)	Presence of essential micronutrients, low oxalate content, 7-day shelf life under refrigeration (4 °C)	[144]
Probiotic strained yogurt-like dessert	Roasted pearl millet flour (RPMF)	Pasteurized milk, RPMF (2.5–8%), powdered sugar (25–45%) and hibiscus extract (2.5–8%)	Improvements in probiotic viability counts; high protein content and elevated levels of antioxidants and essential minerals, with parameters remaining stable for up to 28 days (4 °C)	[145]
Functional goat milk-based nutritional supplement	Fermented pearl millet flour	Freeze-dried goat milk (78%), fermented finger millet and fermented pearl millet flour (1:1) (12%, 24% and 36%), unripe banana powder (8%), date powder (6%), stevia (6%) and cocoa powder (2%). Control–without fermented millet flour	Increased P, Ca, Mg, and Zn levels compared to the commercial reference product.; reduction in vitamins; protein digestibility of 86.04%	[146]
Yogurt	Malted pearl millet flour	Cow milk yogurt and malted pearl millet flour (70:30, 60:40 and 50:50)	Higher Fe, Zn, and P content	[147]
Kibbeh	Roasted millet flour (RM) and wet millet flour (WM)	Formulation 1: beef (85 g), RM (8.5 g), fresh garlic (0.35 g), fresh parsley (0.71 g), fresh mint (1.36 g), salt (1.40 g), soybean oil (2.70 g); Formulation 2: beef (85 g), WM (8.50 g), fresh garlic (0.35 g), fresh parsley (0.71 g), fresh mint (1.36 g), salt (1.40 g), soybean oil (2.70 g), both tested fried and baked	Good oxidation stability during storage for 90 days (−18 °C)	[148]
		With gluten		
Cake	Pearl millet flour through thermoplastic extrusion	Control: Pearl millet extruded flour (250 g), whole-wheat flour (120 g), eggs (230 g), sugar (160 g), soybean oil (160 g), full milk (220 g), sorbitol (28 g), maltitol (28 g), powder yeast (20 g), emulsifier (17 g), calcium propionate (0.3 g), vanilla flavor (14 g); Formulation 1, addition of oat bran (125 g); Formulation 2: addition of toasted rice bran (84 g); Formulation 3, addition of wheat bran (45 g); Formulation 4, addition of psyllium (30 g)	Viable fiber sources while maintaining a soft texture	[33]
Biscuit	Malted pearl millet flour	Control: wheat flour (100%); Formulation ratios: wheat flour (90%, 80%, 70%, 60%), malted pearl millet flour (8%, 16%, 24%, 32%), orange peel flour (2%, 4%, 6%, 8%), added margarine (125 g), sugar (50 g), egg (1 large) and baking powder (3.75 g)	Increase in minerals, protein, and fiber content; increase in hardness, thickness, and diameter; increase in total phenolic content, total flavonoids, vitamin C, and antioxidant activity	[34]
Cookie	Pearl millet flour	Control: wheat (100%); Formulation ratios: pearl millet flour (20%, 40%, 60%, 80%, 100%) added to vegetable ghee, cane sugar, skim milk powder, common salt, sodium bicarbonate, and water	Proportional increase in hardness, chewiness, gumminess, breaking strength, and cutting resistance; proportional increase in fiber and mineral content	[35]
	Pearl millet flour washed with distilled water or alkaline solution (NaOH)	Nine formulations: Control (100% wheat flour) and two sets of wheat substitution (25%, 50%, 75%, and 100%) using H_2_O-washed pearl millet flour and NaOH-washed pearl millet flour	Reduced cookie hardness; increase in phenolic content and antioxidant activity	[36]
Pasta	Pearl millet flour	50:50 blend composition of pearl millet flour and wheat semolina and water	Increase in cooking loss, FFAs, peroxide value, and lipid acidity over storage	[149]
	Pearl millet flour	Blend of wheat semolina and pearl millet flour (100:0, 90:10, 80:20, 70:30, 60:40, 50:50, 40:60, 30:70, 20:80, 10:90, 0:100)	Increasing the pearl millet flour content raised protein, ash, and cooking losses, but reduced hardness, cohesiveness, springiness, gumminess, and chewiness	[150]
Waffles	Pearl millet flour	Control: wheat (100%); wheat, finger millet, and pearl millet flours mixed in the proportions 50:30:20, 50:25:25, 40:40:20; added sugar powder (8 g), milk powder (2 g), sodium bicarbonate (1 g), butter (6 g), wet yeast (2 g), and water (150–155 mL)	Increase in mineral content; decrease in rapidly digestible starch and increase in slowly digestible starch, resistant starch, insoluble dietary fiber and soluble dietary fiber	[151]

**Table 9:** Products elaborated from processed pearl millet grains and their respective evaluations.

Product/ Treatments	Panelists (n)	Sensory Method and Scale	Attributes Evaluated	Findings	Ref.
Brazilian panel					
Whole grain pearl millet-based gluten-free bread	52 regular bread consumers (18–53 years)	10 cm hybrid scale	appearance, color, odor, texture, flavor and overall liking	scores above 5.7 for all attributes and formulations	[152]
	100 consumers (18–70 years)	9-point hedonic scale	overall acceptability	scores above 5.6 for all formulations	[153]
Pasta with raw whole millet and precooked flours by thermoplastic extrusion	86 untrained evaluators	9-point hedonic scale and global acceptance index	taste, texture and appearance and overall acceptability	scores above 5.2 for all attributes; acceptability index ranging between 65% and 68%	[154]
Kibbeh with roasted millet flour and wet millet flour	conducted in two steps: first, 57 untrained volunteers in individual cabins (24–64 years); Second, carried out in a supermarket for three days using 92 potential consumers	9-point hedonic scale	acceptability index, taste, texture, and global appearance	first, acceptability index ranged from 75.4% to 83% for the different samples evaluated; second, tasters rated acceptability well, with mean grades above 6 for all attributes	[148]
Cake with pearl millet extruded flour and different sources of fiber	104 untrained assessors	Check-all-that-apply (CATA) descriptive test, 9-point hedonic scale and 5-point scale for purchase intention	color, aroma, flavor, texture, overall acceptability and purchase intention	scores above 6.2 for all attributes and formulations; purchase intention higher than 3.1 for all cakes	[33]
Other international studies					
Biscuit with finger millet, pearl millet and buckwheat flour	20 semi-trained panelists	9-point hedonic scale	appearance, color, taste, flavor, texture and overall acceptability	scores remained above 7.6 for all attributes after 14 days of storage at room temperature	[140]
Cookies with pearl millet and finger millet	30 untrained members (18–60 years)	5-point hedonic scale	color, taste, odor, texture, and overall acceptability	scores were similar to the control cookie, remaining above 4.5 for all evaluated attributes	[141]
Cookie with pearl millet flour	semi-trained panel (undetermined number of participants)	9-point hedonic scale	color, appearance, flavor, taste, texture and overall acceptability	overall acceptability significantly decreased with increasing levels of pearl millet flour; however, substitution levels up to 60% were found to be more acceptable for their sensory characteristics	[35]
	trained judges (undetermined number of participants)	9-point hedonic scale	appearance, texture, mouthfeel, aroma, taste, aftertaste, and overall acceptability	reduction in scores as the pearl millet flour content increases; acceptable sensory attributes with up to 50% substitution	[36]
Instant beverage with germinated and fermented pearl millet	50 untrained members	9-point hedonic scale	appearance, taste, flavor, mouthfeel, and general acceptability	scores above 6.4 for all attributes	[142]
Instant beverage with pearl millet powder	40 adolescent girls (10–19 years)	9-point hedonic scale	taste/sweetness, color, flavor, sourness, general acceptability	scores above 7.2 for all attributes and 9-month shelf life	[143]
Fermented Beverage with pearl millet fermented flour	35 panelists (18–35 years)	9-point hedonic scale	color, appearance, mouthfeel, flavor, taste, aroma, and overall acceptability	scores above 6.5 for all attributes	[144]
Probiotic strained yogurt-like dessert with roasted pearl millet flour	30 semi-trained panelists	9-point hedonic scale	color and appearance, body and texture and overall acceptability	scores above 6.4 for all attributes and stable for up to 28 days at 4 °C	[145]
Goat milk-based supplement with fermented finger millet and fermented pearl millet flour	15 semi-trained panelists (22–45 years)	8-point hedonic scale	general appearance, color, flavor, consistency, and aftertaste	treatment with 24% millet incorporation showed the highest score in aftertaste and overall acceptability, with scores above 6.0 for all attributes	[146]
Yogurt with malted pearl millet flour	13 panelists	9-point hedonic scale	appearance/color, taste, texture, aroma/smell, mouthfeel, aftertaste, and overall acceptability	scores between 3 and 4 for the evaluated attributes	[147]
Pasta with pearl millet flour	10 untrained members (20–30 years)	9-point hedonic scale	appearance, texture, taste, color and overall acceptability	scores above 6 for all attributes and stable sensory acceptance after 180 days of storage	[149]
Waffles with finger millet and pearl millet flours	25 trained panelists (25–55 years)	appearance/shape, taste and crumb grains/cross section (ranging from 1 to 15); crust color, mouthfeel and softness (ranging from 1 to 10); overall quality < 60, unsatisfactory; 60–65, good; 65–70, very good; 70–75, excellent	appearance/shape, crust color, taste, mouthfeel, softness, crumb grains/cross section	high sensory panel scores and overall quality between 63 and 68 (good/very good)	[151]

## Data Availability

No new data were created or analyzed in this study. Data sharing is not applicable to this article.

## Abbreviations

The following abbreviations are used in this manuscript:

Embrapa	Brazilian Agricultural Research Corporation
FFAs	Free Fatty acids
FAO	Food and Agriculture Organization
Ref.	Reference
SF	Soluble fiber
IF	Insoluble fiber
LDL	Low-Density Lipoprotein
GLP-1	Glucagon-like peptide-1
PYY	Peptide YY
His	Histidine
Ile	Isoleucine
Leu	Leucine
Lys	Lysine
Met	Methionine
Phe	Phenylalanine
Thr	Threonine
Trp	Tryptophan
Val	Valine
ND	Not determined
AMPK	AMP-activated Protein Kinase
SIRT1	Sirtuin 1
PGC-1α	Peroxisome Proliferator-Activated Receptor Gamma Coactivator 1-Alpha
PKA	Protein Kinase A
E-selectin	Endothelial selectin
sICAM	Soluble Intercellular Adhesion Molecule
TLR4	Toll-Like Receptor 4
ROS	Reactive Oxygen Species
DNA	Deoxyribonucleic acid
GAE	Gallic Acid Equivalent
DPPH	2,2-diphenyl-1-picrylhydrazyl
RMF	Raw millet flour
EMF	Extruded millet flour
GMF	Germinated millet flour
TPMF	Milled pearl millet toasted
RM	Roasted millet flour
WM	Wet millet flour
QUE	Quercetin Equivalent
ppm	Parts per million
QE	Quercetin Equivalent
AIN-93G	American Institute of Nutrition-93 growth
PMSGH	Pearl millet starch and germ by dry heat treatment
U2OS	Human Osteosarcoma Cell Line
HFHF	High-fat, high-fructose
TNF-α	Tumor Necrosis Factor alpha
PPARα	Peroxisome Proliferator-Activated Receptor alpha
FVB/n	Friend Virus B-type
HPV	Human Papillomavirus
DMT1	Divalent Metal Transporter 1
Nrf2	Nuclear factor erythroid 2-related factor 2
TNF	Tumor Necrosis Factor
p38 MAPK	p38 mitogen-activated protein kinase
SAPK	Stress-Activated Protein Kinase
JNK	Jun N-terminal Kinase
AKT	Protein Kinase B
LOX	Lipoxygenase
GC-MS	Gas chromatography coupled with mass spectrometry
LC-MS	Liquid chromatography coupled with mass spectrometry
NMR	Nuclear magnetic resonance
LC-HRMS	Liquid Chromatography-High Resolution Mass Spectrometry
ALBP	African locust bean pulp
GF	Gluten free
CAPES	Coordination for the Improvement of Higher Education Personnel
CNPq	Brazilian Research Council
